# A publicly available virtual cohort of four-chamber heart meshes for cardiac electro-mechanics simulations

**DOI:** 10.1371/journal.pone.0235145

**Published:** 2020-06-26

**Authors:** Marina Strocchi, Christoph M. Augustin, Matthias A. F. Gsell, Elias Karabelas, Aurel Neic, Karli Gillette, Orod Razeghi, Anton J. Prassl, Edward J. Vigmond, Jonathan M. Behar, Justin Gould, Baldeep Sidhu, Christopher A. Rinaldi, Martin J. Bishop, Gernot Plank, Steven A. Niederer

**Affiliations:** 1 School of Biomedical Engineering and Imaging Sciences, King’s College London, London, City of London, United Kingdom; 2 Institute of Biophysics, Medical University of Graz, Graz, Steiermark, Austria; 3 IHU Liryc, Electrophysiology and Heart Modeling Institute, fondation Bordeaux Université, F-33600 Pessac- Bordeaux, France; 4 University of Bordeaux, IMB, UMR 5251, F-33400 Talence, France; 5 Guy’s and St Thomas’ NHS Foundation Trust, London, City of London, United Kingdom; 6 NumeriCor GmbH, Graz, Austria; Beijing University of Technology, CHINA

## Abstract

Computational models of the heart are increasingly being used in the development of devices, patient diagnosis and therapy guidance. While software techniques have been developed for simulating single hearts, there remain significant challenges in simulating cohorts of virtual hearts from multiple patients. To facilitate the development of new simulation and model analysis techniques by groups without direct access to medical data, image analysis techniques and meshing tools, we have created the first publicly available virtual cohort of twenty-four four-chamber hearts. Our cohort was built from heart failure patients, age 67±14 years. We segmented four-chamber heart geometries from end-diastolic (ED) CT images and generated linear tetrahedral meshes with an average edge length of 1.1±0.2mm. Ventricular fibres were added in the ventricles with a rule-based method with an orientation of -60° and 80° at the epicardium and endocardium, respectively. We additionally refined the meshes to an average edge length of 0.39±0.10mm to show that all given meshes can be resampled to achieve an arbitrary desired resolution. We ran simulations for ventricular electrical activation and free mechanical contraction on all 1.1mm-resolution meshes to ensure that our meshes are suitable for electro-mechanical simulations. Simulations for electrical activation resulted in a total activation time of 149±16ms. Free mechanical contractions gave an average left ventricular (LV) and right ventricular (RV) ejection fraction (EF) of 35±1% and 30±2%, respectively, and a LV and RV stroke volume (SV) of 95±28mL and 65±11mL, respectively. By making the cohort publicly available, we hope to facilitate large cohort computational studies and to promote the development of cardiac computational electro-mechanics for clinical applications.

## Introduction

In the last decades, computational models for cardiac electro-mechanics underwent a rapid development. The increased level of detail included in such models have made them more appealing to the clinical community. Significant progress has been made in the methods and software to simulate single hearts. However, simulating cardiac electro-mechanics on large cohorts still remains a challenge.

Previous cohorts of personalised multi-scale models were focused on one or two cardiac chambers. One of the largest computational studies used forty-six biventricular meshes to simulate ventricular electrical activation and mechanical contraction [1]. The results of the personalised models were then analysed to find novel bio-marker candidates to improve disease severity stratification. Single-patient models [2, 3] and smaller cohorts of biventricular meshes [3–10] also proved the potential of computational modelling for predicting and planning patient treatment, and improving our understanding of cardiac physiology and pathophysiology. Small patient cohorts, however, have a limited capability to capture patient anatomical variability. Furthermore, biventricular models have limitations in representing realistic systolic motion, as anatomical cardiac structures surrounding the ventricles are not included [11].

Recently, computational electro-mechanics started moving towards four-chamber models [11–14]. Simulations including the atria as well as the major vessels allow for a more realistic predicted motion, therefore offering an increased predictive power of the model. Furthermore, the interest in the role of atrial dynamics on ventricular function has been growing in the clinical community [14–16]. Four-chamber heart models are, however, still in their infancy, as existing studies focus on one single heart rather than a cohort of individuals [12–14, 17, 18].

As previously discussed, capturing patient anatomical variability is fundamental for computational electro-mechanics models intended for clinical applications. Representing variation in anatomy across a cohort of individuals is, however, a well-known challenge in medical imaging analysis. In the last decade, cardiac atlases were increasingly used to study variability in shape [19–23] and motion [24–28] in healthy and diseased states. As with simulation studies, most of these atlases are focused on one cardiac chamber. In 2013, Hoogendoorn et al. published an atlas of four-chamber heart models based on 138 subjects [29]. The atlas was made publicly available, and constitutes a powerful tool for the study of population variability and for electro-mechanics computational studies, as it can be registered and warped to available medical images to generate a patient-specific geometry. However, the atlas is made of a surface and not a volumetric mesh and represents summary statistics for a population and not specific meshes of individuals. Generating a patient specific mesh from these data requires medical images, meshing tools to move from a surface to a volumetric mesh and labelling of surfaces and regions to impose spatial varying properties and boundary conditions.

In this paper, we build the first publicly available cohort of four-chamber heart models generated from twenty-four heart failure (HF) patients. We develop a semi-automatic pipeline to build three-dimensional tetrahedral meshes of the whole heart from CT images. We test the usability of the meshes for cardiac electro-mechanics simulations by running electro-mechanical simulation tests on all twenty-four meshes. To facilitate the development of new simulations and model analysis techniques by groups without direct access to medical data, image analysis techniques and meshing tools we make the cohort of meshes available for download (DOI 10.5281/zenodo.3890034 [30]).

## Materials and methods

We first describe the image acquisition protocol and the data available for each patient. The pipeline we built to generate four-chamber segmentation’s from high-resolution CT images is then presented, followed by the meshing process and ventricular fibres assignment. Finally, we describe the electro-mechanics simulation tests we used to demonstrate that our meshes are suitable for electro-mechanics simulations.

### Ethics statement

Data were gathered as part of two clinical trials: clinical trial REC numbers 14/WM/1069 and 18/LO/0752 approved by the West Midlands Coventry & Warwick ethics committee and by the London-Harrow ethics committee, respectively. Data were analysed anonymously.

### Clinical data

Our models were based on twenty-four cardiac resynchronization therapy (CRT) patients recruited for CRT upgrade. Demographics of the cohort are shown in Table 1. Patients underwent ECG-gated CT acquired in 10 or 20 frames over an entire cardiac cycle prior to the CRT upgrade procedure. CT images resolution for each patient is shown in the last column of Table 1. For one patient (case 03), only the ED frame was acquired. Baseline conditions were assessed with 12-lead ECG (QRS duration shown in column 4, Table 1) and ejection fraction (EF) measured with 2D echo (Table 1, column 5). For patients 11 and 22, reported EF was measured with CT because EF echo measurements were not available.

**Table 1:** Patients cohort demographics.

	Age [y]	Sex [-]	QRSd [ms]	EF [%]	PP [mmHg]	EDP [mmHg]	dP/dt_max_ [mmHg ⋅ s^-1^]	dP/dt_min_ [mmHg ⋅ s^-1^]	CT resolution [mm×mm×mm]
**patient01**	83	M	176	23	119	15	934	-1342	0.44×0.44×1.0
**patient02**	66	M	168	26	126	17	905	-896	0.49×0.49×0.4
**patient03**	72	M	178	36	96	18	899	-1016	0.42×0.42×0.4
**patient04**	49	M	152	23	92	21	608	-938	0.48×0.48×0.4
**patient05**	62	M	134	17	130	25	688	-795	0.49×0.49×0.4
**patient06**	85	M	152	32	90	9	686	-779	0.38×0.38×0.4
**patient07**	37	M	186	41	113	22	920	-992	0.38×0.38×0.5
**patient08**	79	M	200	41	125	21	1092	-1078	0.49×0.49×0.4
**patient09**	40	M	180	42	73	7	592	-988	0.41×0.41×0.4
**patient10**	76	M	180	41	100	11	1131	-1339	0.44×0.44×0.5
**patient11** **	76	M	144	54*	-	-	-	-	0.42×0.42×0.4
**patient12**	69	M	140	15	100	20	620	-702	0.38×0.38×0.5
**patient13**	72	M	134	35	110	25	829	-865	0.45×0.45×0.5
**patient14** **	61	M	180	20	-	-	-	-	0.43×0.43×0.5
**patient15**	49	M	182	28	94	4	1201	-1756	0.39×0.39×0.5
**patient16**	55	M	134	47	114	8	1600	-1333	0.40×0.40×0.5
**patient17**	76	M	120	40	93	14	942	-937	0.45×0.45×0.5
**patient18**	82	M	132	31	178	9	1343	-1478	0.32×0.32×0.5
**patient19** **	68	M	131	25	-	-	-	-	0.37×0.37×0.5
**patient20** **	71	M	145	37	-	-	-	-	0.37×0.37×0.5
**patient21**	72	M	141	42	112	13	1144	-1252	0.39×0.39×0.5
**patient22**	53	M	161	36*	133	11	1126	-1134	0.43×0.43×0.5
**patient23**	79	F	186	40	171	17	1282	-1638	0.33×0.33×0.4
**patient24**	84	M	174	43	70	-3	873	-999	0.43×0.43×0.4
	67±14	-	159 ± 23	34 ± 10	112 ± 28	14 ± 7	971 ± 270	-1113 ± 290	

*EF measured with CT and not 2D echo as echo data were not available

**Invasive pressure recordings not available

LV pressure was recorded invasively during the upgrade procedure. For each patient, we processed baseline pressure measurements (acquired at the beginning of the upgrade procedure, before pacing) by averaging the acquired trace over five to ten heart beats. Ectopic beats were manually identified and discarded. We were therefore able to measure the baseline peak in systolic pressure, ED pressure, dP/dt_max_ and dP/dt_min_. There data are shown in columns 6 to 9 of Table 1. For four patients (cases 11, 14, 19 and 20), pressure data were not available. We note that the end diastolic pressure for case 24 is -3mmHg. This is clearly not the absolute pressure but potentially reflects artefacts due to either respiration or that calibration occurs outside the patient.

### Segmentation

**Fig 1:**
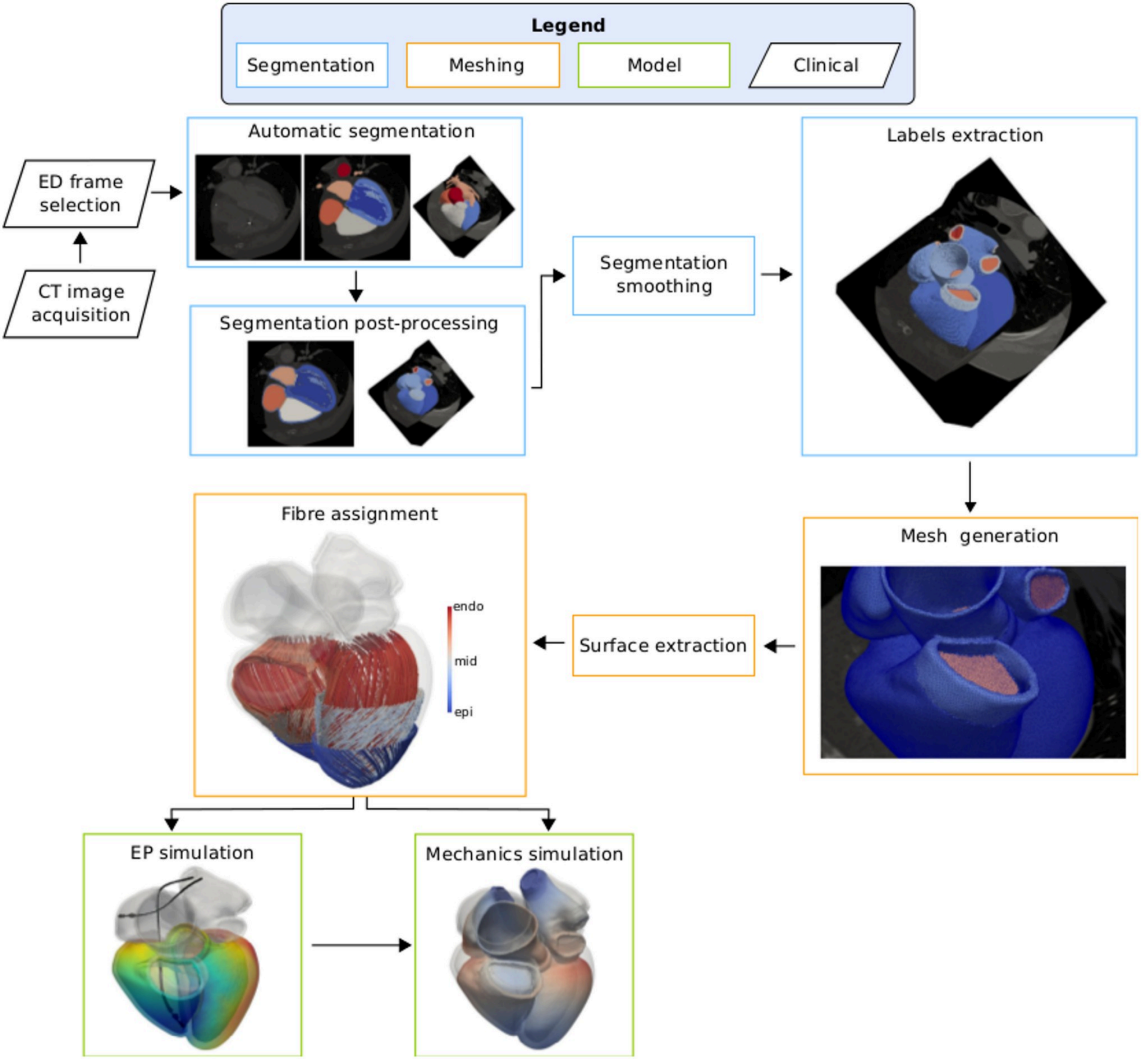
Pipeline. CT images were acquired in 10 or 20 frames over a whole cardiac cycle. The end-diastolic (ED) geometry was then selected and automatically segmented [31]. The resulting segmentation was post-processed to generate labels representing the RV, LA and RA myocardium, the wall thickness for the aorta and the pulmonary artery, valve planes and rings at the cropped veins. The surface of the segmentation was then smoothed [37] and all the labels but the blood pools and the LV papillary muscles were extracted to generate a tetrahedral mesh. We extracted the endocardial and epicardial surfaces of the atria and ventricles and we assigned fibre orientation to the ventricles with a rule-based method [38]. Finally, we simulated electrical activation and mechanical contraction of the ventricles to test the usability of the meshes.

We segmented ED CT images with a semi-automatic pipeline shown in Fig 1. An automatic segmentation method was used to generate labels representing the LV blood pool, LV trabeculations and papillary muscles, LV myocardium, and right ventricular (RV), left atrial (LA), right atrial (RA) and aorta blood pools [31]. The resulting segmentation was post-processed with the free software Seg3D [32]. For cases 13 and 21, the automatic segmentation failed to precisely segment the LV myocardium. Therefore, the LV papillary muscles and the LV blood pool were combined and the resulting region dilated until the segmentation visually matched the epicardium of the LV free wall. In all cases the RV, LA, RA and aortic walls could not be automatically segmented. To estimate the RV wall, the RV blood pool was dilated by 3.5mm, based on values for RV wall thickness reported in the literature [33, 34]. The same procedure was applied to the blood pools of the LA, the RA and the aorta to generate labels representing the LA myocardium, the RA myocardium and the wall thickness of the aorta, with a wall thickness of 2mm [35, 36]. This procedure sometimes caused the labels to overlap. For each of the overlapping regions, the region that was best captured by the image was prioritised. The other label was modified by locally dilating it towards the corresponding blood pool. The LV blood pool, the LV myocardium, the aortic blood pool and wall thickness had highest priority as the automatic segmentation tool performed a better segmentation of these labels. Therefore, those regions were never modified. If any of the other labels overlapped with those, it was adjusted to prevent overlap. In case of overlap between the LA and the RA, the LA blood pool and myocardium were prioritised over the RA, as the image contrast of the LA blood pool was better compared to the contrast at the RA blood pool. The LA segmentation was not modified, while the RA myocardium was adjusted to maintain a constant RA wall thickness.

The pulmonary veins, the superior vena cava and the inferior vena cava were cut from the LA and the RA blood pool perpendicular to the vessel direction. Rings at each of the cut veins were generated to apply boundary conditions in the mechanics simulation. The left atrial appendage (LAA) was only partially covered by the CT image volume for some of the cases. For consistency throughout the cohort, we cropped the LAA from the LA blood pool as we did for the pulmonary veins. Labels representing a small portion of the pulmonary artery blood pool and wall thickness were generated by cutting the RV blood pool and myocardium at the ouflow tract, perpendicular to the vessel direction. The endocardial surface of atria and ventricles were closed with 2mm-thick valve planes at each inflow and outflow [14, 39]. Although this constitutes a simplification of the complex structure of the four cardiac valves, anatomical details of the valve leaflets and of the chordae tendinae of the mitral and tricuspid valves were not visible in the CT images. Therefore, we could not render such structures in the final segmentation.

In order to reduce the staircase effect due to image artifacts or small manual corrections, the resulting four-chamber segmentation was up-sampled and smoothed with an isotropic resolution of 150*μ*m with a variational approach [37]. Prior to the mesh generation step, labels for the LV, RV, LA, RA myocardium, wall thickness of the aorta and the pulmonary artery, all the valve planes and all the rings generated at the cut veins were extracted. We show the segmentation for the twenty-four cases in S1–S6 Figs in the Supporting Information.

Although LV and RV myocardium show intricate trabeculation, we did not include these structures in the segmentation we used to generate the final meshes. Some LV trabeculations and the papillary muscles were grossly segmented by the automatic segmentation tool, but the resolution of the CT images was not high enough to capture them completely. RV trabeculations were not visible in the CT images. Therefore, we excluded these structures from the segmentation and we meshed only the smooth myocardial wall.

### Mesh generation

**Fig 2:**
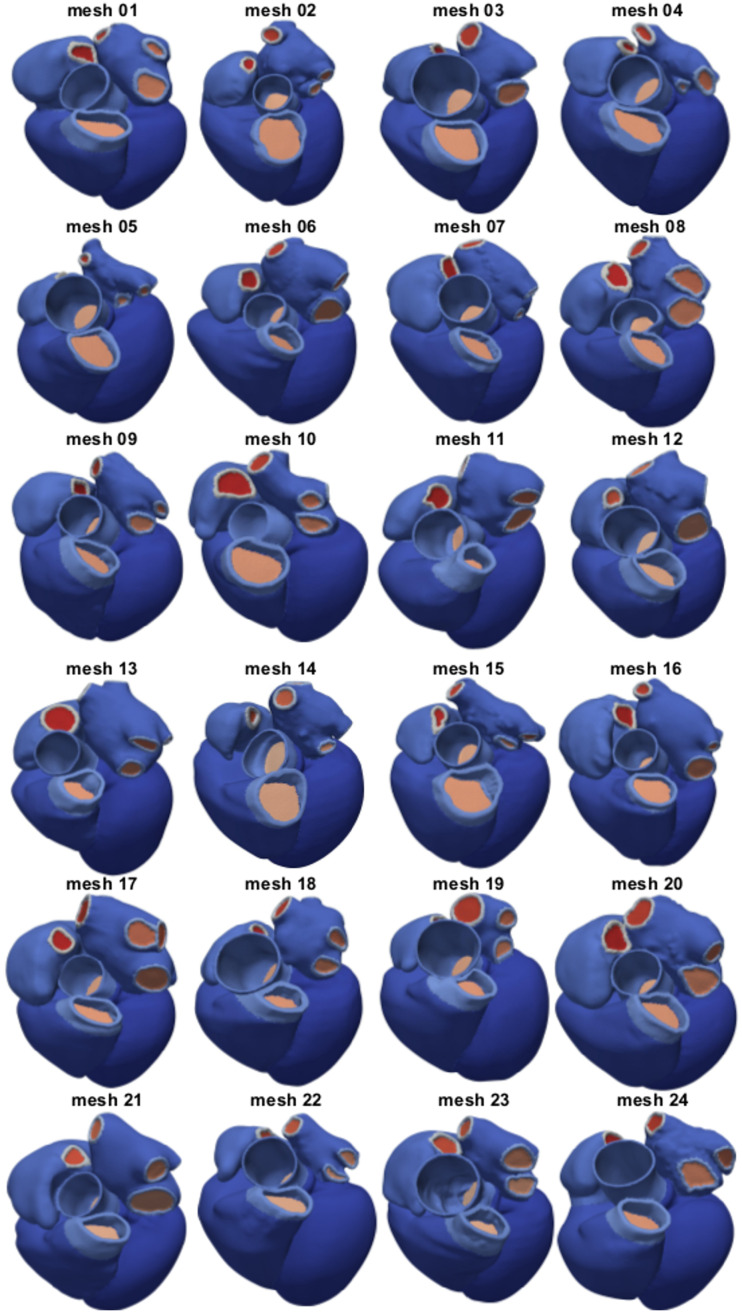
Twenty-four four-chamber heart meshes. The images show an anterior view of the twenty-four meshes generated with the pipeline described in the text.

**Fig 3:**
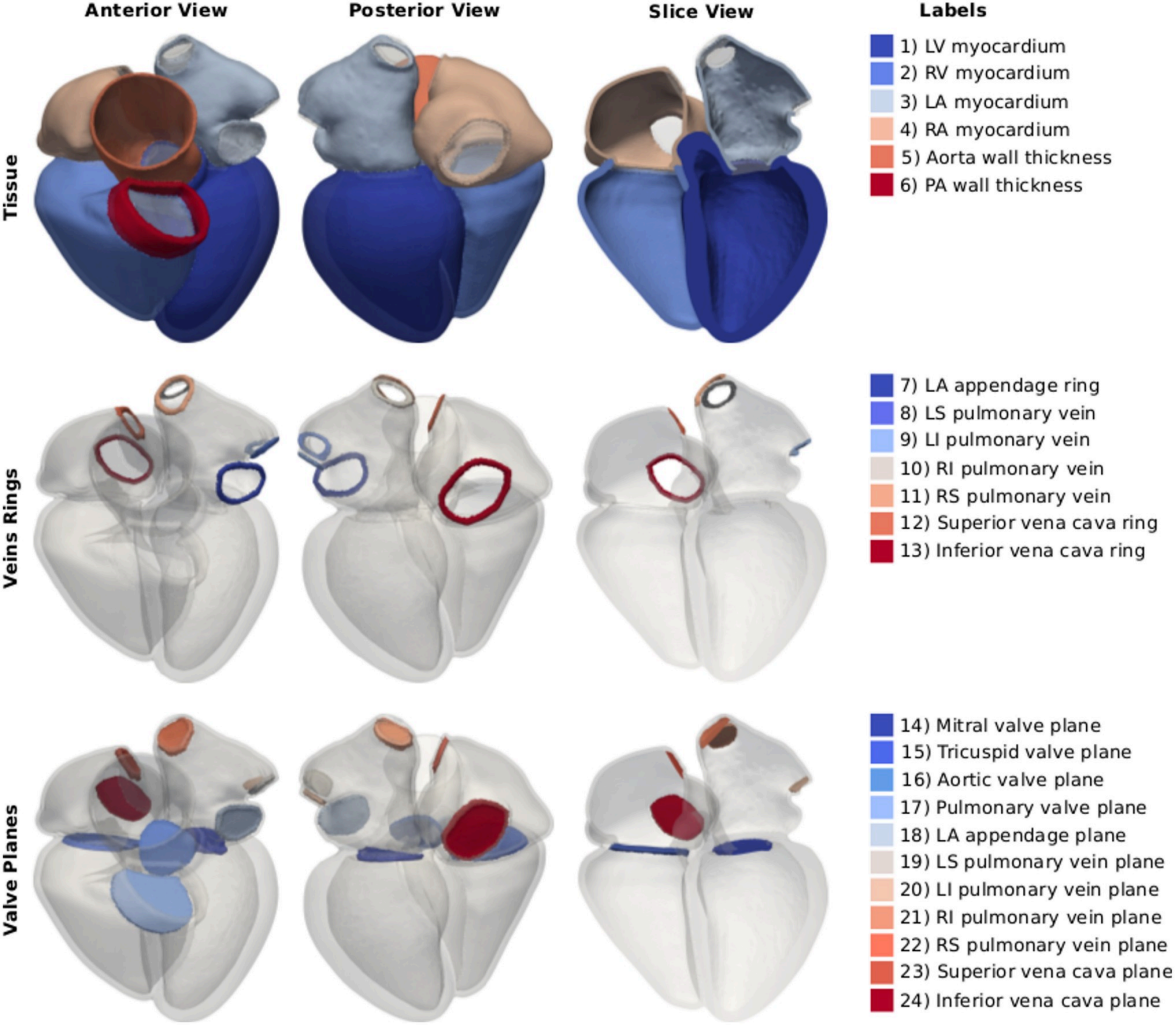
Labels description. The images on the left and in the center show an anterior and a posterior view of one of the four-chamber meshes. The image on the right shows an anterior view of a clip of the geometry. On the right, the twenty-four labels of the mesh are listed. The first row shows the myocardium of the LV, RV, LA, RA and the wall of the cropped aorta and pulmonary artery (PA). The second row shows the rings at the cropped veins and at the LAA. The third row shows the valve planes added at all inlets and outlets of the LV, RV, LA and RA.

We used the multi-label segmentation to generate tetrahedral meshes with a target resolution of 1mm using CGAL (Computational Geometry Algorithm Library). Fig 2 shows the resulting twenty-four four-chamber heart meshes. Label numbering is shown in Fig 3. The first four labels represent the myocardium of the LV, RV, LA and RA, respectively. Labels five and six correspond to the wall of the aorta and the pulmonary artery. Labels from seven to eleven represent the rings of the cropped LAA and of the four pulmonary veins. Labels twelve and thirteen are the rings at the cropped superior and inferior vena cava. The mitral, tricuspid, aortic and pulmonary valve planes correspond to labels from fourteen to seventeen, while labels from eighteen to twenty-four represent planes for the cut veins, in the same order of the rings. Mesh 12 and 14 had only three pulmonary veins, as the branching between left superior and inferior pulmonary veins occurred further away from the LA and was therefore not covered by the CT image volume. The resulting meshes therefore have only twenty-two labels instead of twenty-four.

Fibre and sheet directions in the ventricles were assigned using a rule-based method [38]. The base of the ventricles was defined as the common surface nodes between the ventricles and the mitral and the tricuspid valve planes. The endocardium and the epicardium of the ventricles were extracted as the surface nodes separated by the base. Ventricular endocardium and epicardium were assigned with a fibre orientation of 80° and -60°, respectively [14, 39]. For the sheet direction, we used -65° at the endocardium and 25° at the epicardium [38]. Although myocardial microstructure was reported to change in heart failure [40], this was not accounted for by the used rule-based method. The fibre and sheet directions represent those of a healthy human heart.

**Fig 4:**
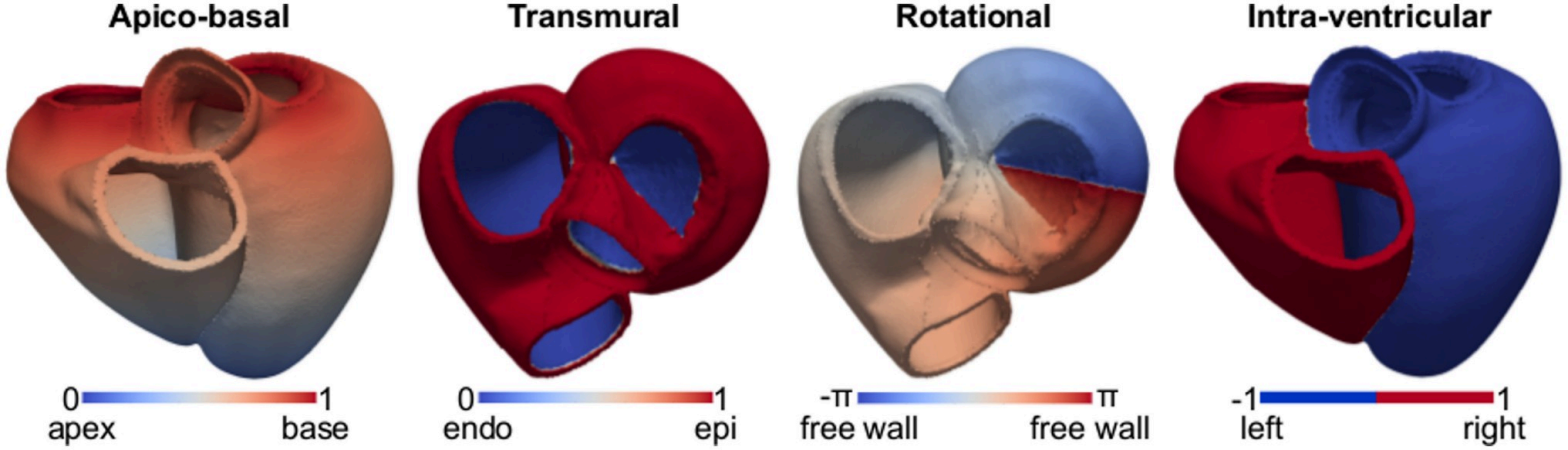
Universal ventricular coordinates. From left to right: apico-basal coordinate, ranging between 0 at the apex and 1 at the base (anterior view); transmural coordinate, varying from 0 at the endocardium of the LV and of the RV free wall to 1 at the epicardium (top view); rotational coordinate, ranging between −*π* at the LV free wall, to 0 at the septum and *π* back to the LV free wall; intra-ventricular coordinate, defined at -1 at the LV and +1 at the RV.

To have a common frame of reference for the twenty-four meshes, we applied a universal ventricular coordinate (UVC) algorithm to all the geometries [41]. This resulted in a system of four coordinates for the ventricles of each mesh, shown in Fig 4: 1) apico-basal coordinate ranging from 0 at the LV apex to 1 at the base; 2) rotational coordinate rotating around the LV, ranging from −*π* at the LV free wall, 0 at the septum and *π* back to the LV free wall; 3) a transmural coordinate, ranging from 0 at the endocardium to 1 at the epicardium; 4) an intraventricular coordinate, defined at -1 at the LV +1 at the RV. We assigned ventricular nodes of the meshes with a value for each of these four coordinates. Non-ventricular nodes were assigned with a value of -100.

### Mesh refinement

Although an average 1mm edge length is enough to solve for electrical activation times with simplified models (e.g. eikonal), a much finer resolution is needed for more complex models (e.g. monodomain or bidomain). To show that it is possible to refine the given meshes, we resampled all twenty-four original 1mm-resolution meshes to achieve a 0.35mm resolution using *meshtool* (https://bitbucket.org/aneic/meshtool/src/master/). These high resolution meshes will also be publicly available as part of the database, inclusive of element tags as described above (Fig 3) and ventricular fibres. If the user desires an even higher resolution, the finer meshes provided in the database can be further refined using *meshtool*.

### Database format

We provide a zipped folder for each mesh on zenodo (DOI 10.5281/zenodo.3890034 [30]). Each folder contains the coarse and the finer versions of the same mesh. All twenty-four 1mm-meshes are supplied in *case* format, readable with *paraview* (https://www.paraview.org/). All binary files containing the meshes data (*ens* and *geo* formats) are provided within the zipped folder. Points coordinates are given in mm. Element tags are assigned to the elements of the mesh (see Fig 3), as well as fibres and sheet directions. Fibres and sheet directions are assigned to the ventricles according to the rule-based method explained above. Non-ventricular elements are assigned with default vectors [1, 0, 0] and [0, 1, 0]. UVCs are assigned to the nodes of the meshes. Non-ventricular nodes are assigned to value of -100 for all four UVCs. We also provide the location of the CRT right-ventricular electrode used to initiate ventricular excitation. This is given as a label on the nodes called *electrode_endo_rv*, which is 1 at the stimulated nodes. Finer meshes are provided in *vtk* format, also readable in *paraview*. For these meshes, we provide element tags, fibres and sheet directions on the ventricles, all in the same file.

### Electro-mechanics simulation framework

To ensure that that our mehes are suitable for electro-mechanics simulations, we ran electro-mechanics simulation tests on all twenty-four 1mm-resolution meshes using linear tetrahedral elements. We simulated ventricular electrical activation and free mechanical contraction.

**Fig 5:**
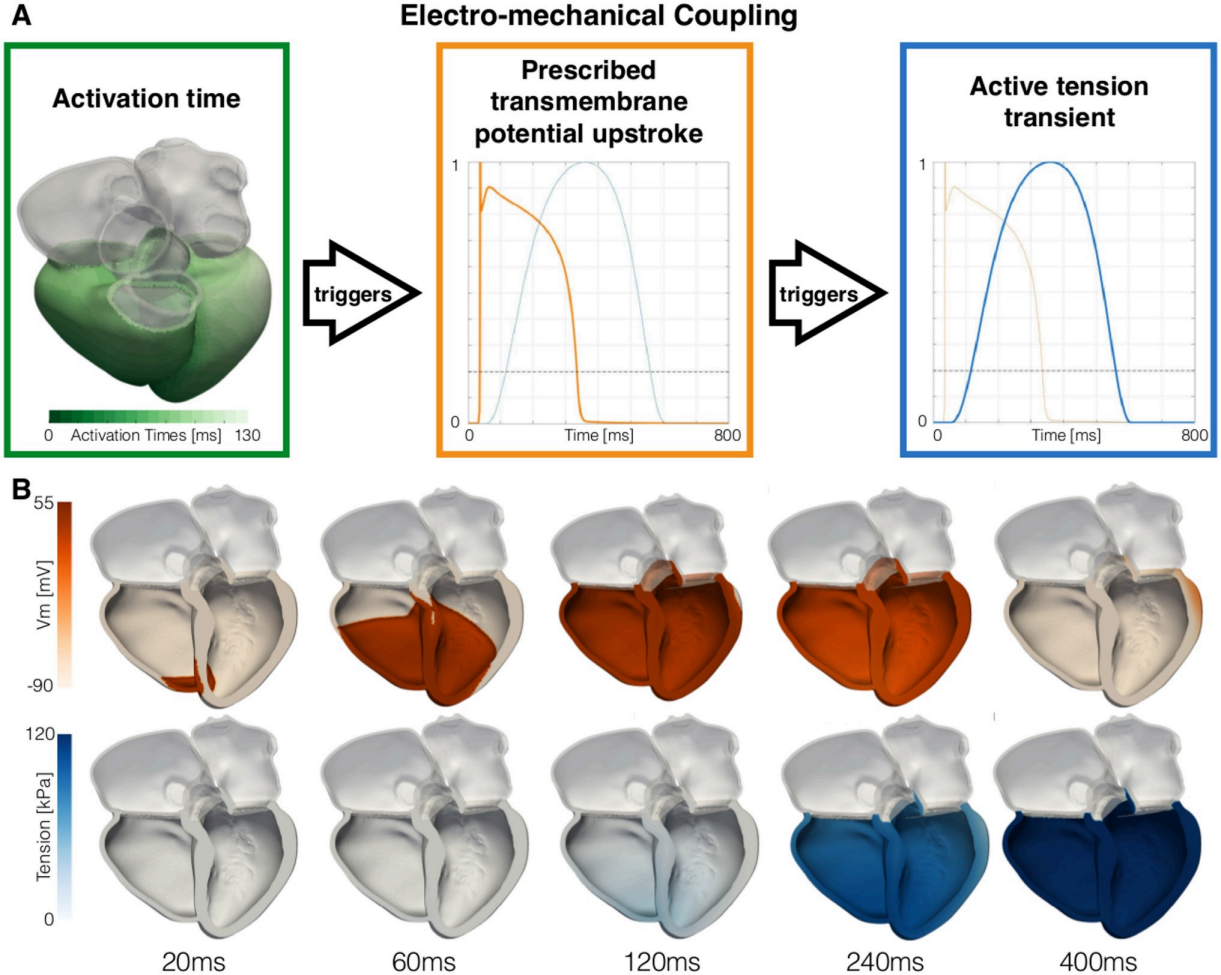
Electro-mechanical coupling. **A** The activation times computed by the eikonal model (shown on mesh 01 in the green box) trigger an upstroke in transmembrane potential V_m_ at each node of the mesh (orange curve in the orange box). When the transmembrane potential overcomes -60mV (black dashed line), the rise in active tension is triggered (blue curve in the blue box). **B** We show the changes in transmembrane potential (top row) and the active tension (bottom row) over time on mesh 01.

Ventricular electrical activation was simulated with a reaction-eikonal model [42]. Ventricular myocardium was assigned with a longitudinal and transverse conduction velocity of 0.6m s^−1^ and 0.24 s^−1^, respectively, consistently with experimental measurements on human ventricular tissue [43]. To simulate early activation of ventricular endocardium, we added a one-element thick endocardial layer to both ventricles and we increased its conduction velocity by two fold [6, 44]. The RV endocardium was stimulated based on the location of the RV lead, segmented by thresholding the ED CT image. The radius of the stimulus site was set to 5mm to approximate the area covered by the tip of the CRT lead. We simulated only one beat for each heart. The RV endocardium was therefore stimulated only once at 0.0ms with a stimulus duration of 2.0ms. To simulate left bundle branch block activation typical of patients with dyssynchrony, the LV was not stimulated [45]. We simulated transmembrane potential changes due to electrical activation using a reaction-eikonal model with diffusion [42]. The activation time computed by the eikonal model triggers the upstroke in transmembrane potential V_m_, shown in orange in Fig 5A. As the transmembrane potential overcomes a threshold set at -60mV, the rise in active tension is triggered. Ventricular action potential was determined using the Ten-Tusscher model [46]. As our main interest was in activation time and not reporalisation we used a homogeneous epicardial cell type across the myocardium.

Large deformations of the heart throughout the cardiac cycle were modelled with the finite elasticity equations solved in a Lagrangian reference frame [47]. Cardiac tissue was modelled as a hyperelastic and nearly incompressible material. For ventricular passive mechanics we used the transversely isotropic Guccione law [48]:

$$\begin{array}{cc} \Psi (\text{E}) & =\frac{C}{2}(e^{Q}-1)+\frac{\kappa }{2}{\text{ln}}^{2}\left(J\right)\;,\\ Q & =b_{ff}E_{ff}^{2}+2b_{fs}\left(E_{fs}^{2}+E_{fn}^{2}\right)+b_{ss}\left(E_{ss}^{2}+E_{nn}^{2}+2E_{sn}^{2}\right)\;, \end{array}$$

where Ψ is the strain energy function and *C*, *b*_*f*_, *b*_*fs*_ and *b*_*ss*_ are the material parameters. The *f*, *s* and *n* in the Cauchy-Green strain tensor **E** represent the strain in the fibres, sheet and normal to sheet directions, while *J* indicates the Jacobian determinant of the deformation gradient tensor. Parameters for ventricular stiffness were set based on parameters estimated in HF patients [49] and are shown in Table 2. Passive mechanics of non-ventricular tissue was modelled with a neo-Hookean model to represent non-linear response of the material to external load:

$$\begin{array}{c} \Psi \left(\text{E}\right)=c\left(I_{1}-3\right)+\frac{\kappa }{2}{\text{ln}}^{2}\left(J\right)\;, \end{array}$$

where *c* is the only material parameter and *I*_1_ denotes the first invariant of the Cauchy-Green strain tensor. For atrial myocardium, wall of the aorta, pulmonary artery and veins, we ignored the effect of the fibres, and we set passive stiffness parameters using the following values from the literature. For the atria, we used the average of the parameter values of the anterior and posterior regions reported in the literature [50], resulting in c = 7.45kPa. For the aorta and the pulmonary artery, we imposed c = 26.66kPa and c = 3.7kPa, the average parameter value of the two cases reported in previous works, respectively [51, 52]. Rings at the cropped pulmonary veins, superior vena cava, inferior vena cava and LAA were assigned with the same parameter value used for the atria. To restrict their deformation, the valve planes were assigned with a parameter c = 1000.0kPa. Tissue incompressibility was enforced with a penalty method [53, 54], with a bulk modulus *κ* = 1MPa.

**Table 2:** Passive and active mechanics material parameters.

**Passive Material Parameters**					
	C	b_f_	b_fs_	b_t_	*κ*
	kPa	-	-	-	MPa
Ventricles	3.0	19.25	8.75	7.0	1.00
	c				k
	kPa				MPa
Atria	7.45				1.00
Aorta	26.66				1.00
Pulmonary artery	3.7				1.00
Valve planes	1000.0				1.00
Cropped veins & LAA	7.45				1.00
**Active Tension Parameters**					
	T_peak_	t_emd_	t_dur_	*τ*_r_	*τ*_d_
	kPa	ms	ms	ms	ms
Ventricles	125	20	550	130	100

Active tension transient of ventricular myocardium was simulated with a phenomenological model [2, 55], discarding the effect of length-dependence. Active tension *T*_*a*_ was computed as follows:

$$\begin{array}{cc} T_{a} & =T_{\text{peak}}{\text{tanh}}^{2}(\frac{t}{\tau_{r}})\;{\text{tanh}}^{2}\;(\frac{t_{\text{dur}}-t}{\tau_{d}}),\;0<t_{s}<t_{\text{dur}}\;,\\ t_{s} & =t-t_{\text{act}}-t_{\text{emd}}\;. \end{array}$$

where T_peak_ is the peak in active tension, t is time and t_act_ is the activation time computed with the eikonal model. Electro-mechanical delay t_emd_, duration of the twitch t_dur_, rising time *τ*_r_ and decay time *τ*_d_ were set to 20ms, 550ms, 130ms and 100ms, respectively. T_peak_ was set to 125.0kPa to achieve an LV EF of about 35% for all cases. This falls within the range of EF measured in our cohort (34±10%). The active tension transient is shown in Fig 5A (blue curve). We assumed that active contraction occurs only in the fibre direction. Therefore, the active stress computed by the cellular model was added to the passive stress in the fiber direction only [47]. Including transverse active stress was reported to improve simulated strains [56], but was shown to have minimal effects on simulated motion [11]. However, the aim of active contraction test was not to match physiological strains but to show the suitability of the meshes for electro-mechanics simulations. All parameters used for passive and active mechanics are shown Table 2.

We wanted to verify that our meshes element quality was high enough to endure a more significant contraction, with a left ventricular ejection fraction falling within healthy ranges (48%-69%) [57]. Since the meshes of our cohort were generated from HF patients, some ventricles are very dilated with LV EDV sometimes exceeding 400mL. For dilated meshes with left ventricular end-diastolic volume above normal values (>232mL [57]), a 60% ejection fraction would require abnormally large stroke volume between 161mL and 262mL, while normal values range between 59mL and 132mL [57]. For only twelve of our meshes, LV EDV falls within normal and border zone values between 109mL and 232mL, from the UK Biobank database of 804 cases [57]. For these meshes, we ran additional simulations with decreased passive ventricular stiffness to healthy values available in the literature [49]: C = 1.7kPa, b_f_ = 8.0, b_fs_ = 4.0 and b_t_ = 3.0. Parameters for electrical activation and active contraction were left unchanged.

#### Boundary conditions

The mechanics simulation was constrained by applying omni-directional springs at the right superior and inferior pulmonary veins, and at the superior vena cava [14, 18], with a spring stiffness of 10kPa mm^−1^. The effect of the pericardium on the ventricles was simulated by applying unidirectional springs to the epicardium to penalise displacement normal to the surface, using the ED state as the reference configuration for the springs [18]. We used a map to scale the penalty for the displacement of ventricular epicardium normal to the surface. The map was based on the average image-derived displacement for all patients. Details about how the penalty map was computed are provided in S2 Appendix.

We did not account for ventricular preload or ventricular afterload. The initial configuration was assumed to be ventricular end-diastole, and we did not perform any unloading of the mesh. The ventricles were assumed to contract against zero pressure and zero resistance. Therefore, we did not include any representation of the aortic and pulmonary valves. The atria were passive, with no electrical excitation and active mechanical contraction. Our model did not include any representation of flow across the atrio-ventricular valves.

#### Numerical methods

All the electro-mechanical simulations were run with the Cardiac Arrhythmia Research Package (CARP) [12, 58, 59]. The eikonal equation was solved with the fast iterative algorithm [41, 60]. The reaction-eikonal model used to compute transmembrane potential distribution has been described previously [42].

For details of the finite element discretisation of the mechanics equations, we refer to Augustin et al. [61]. The mechanics solver we used was validated with benchmark tests [62].

## Results

First, we present results for the general characteristics of the twenty-four meshes. Then, we present results for the electro-mechanics simulations. Finally, we investigate the relationship between LV and RV anatomy and simulation outputs.

### Mesh characteristics

To test mesh quality we computed the scaled Jacobian for each element of each mesh to have a measure of the distortion of the tetrahedra [63, 64]. A scaled Jacobian close to 1 indicates a perfectly regular tetrahedron, while a scaled Jacobian close to 0 indicates a highly distorted tetrahedron. Table 3 shows the results for mesh quality for the twenty-four meshes, together with the number of nodes, number of elements and average edge length. We show results for both the coarse and the high resolution meshes.

**Table 3:** Meshes characteristics. The table shows the number of nodes, number of elements, average element quality (measured as the scaled Jacobian) and edge length for the twenty-four meshes. For the element quality and the edge length, we also reported the standard deviation within each mesh in brackets.

	Coarse meshes				High-resolution meshes			
	# nodes	# elements	tet quality	edge length [mm]	# nodes	# elements	tet quality	edge length [mm]
**mesh01**	481066	2349414	0.74 (0.11)	1.06 (0.15)	9745931	48831236	0.51 (0.13)	0.39 (0.10)
**mesh02**	691916	3490090	0.74 (0.11)	1.06 (0.15)	14351113	72647039	0.51 (0.13)	0.39 (0.10)
**mesh03**	527572	2636456	0.74 (0.12)	1.07 (0.15)	10887723	54949789	0.51 (0.13)	0.39 (0.10)
**mesh04**	563509	2873599	0.74 (0.11)	1.06 (0.15)	11772640	59745743	0.52 (0.13)	0.39 (0.10)
**mesh05**	637354	3266530	0.74 (0.11)	1.06 (0.15)	13356834	67888242	0.52 (0.13)	0.39 (0.10)
**mesh06**	539616	2656723	0.73 (0.11)	1.06 (0.15)	10994447	55305234	0.51 (0.13)	0.39 (0.10)
**mesh07**	529708	2598475	0.73 (0.11)	1.06 (0.15)	10777223	54166600	0.51 (0.13)	0.39 (0.10)
**mesh08**	502241	2489060	0.74 (0.11)	1.06 (0.15)	10286788	51801290	0.51 (0.13)	0.39 (0.10)
**mesh09**	451528	2227614	0.74 (0.11)	1.06 (0.15)	9203067	46286710	0.51 (0.13)	0.39 (0.10)
**mesh10**	445112	2176295	0.74 (0.11)	1.06 (0.15)	9014841	45230037	0.51 (0.13)	0.39 (0.10)
**mesh11**	440291	2169085	0.74 (0.11)	1.06 (0.15)	8963833	45070335	0.51 (0.13)	0.39 (0.10)
**mesh12**	536011	2635144	0.73 (0.11)	1.06 (0.15)	10909862	54838853	0.51 (0.13)	0.39 (0.10)
**mesh13**	562910	2788537	0.74 (0.11)	1.07 (0.15)	11546318	58139068	0.51 (0.13)	0.39 (0.10)
**mesh14**	674011	3423028	0.74 (0.11)	1.06 (0.15)	14028329	71143503	0.52 (0.13)	0.39 (0.10)
**mesh15**	573966	2866740	0.74 (0.11)	1.06 (0.15)	11794981	59534694	0.51 (0.13)	0.39 (0.10)
**mesh16**	490365	2360470	0.73 (0.11)	1.07 (0.15)	9862653	49283398	0.51 (0.13)	0.39 (0.10)
**mesh17**	572656	2807390	0.73 (0.11)	1.07 (0.15)	11665496	58599804	0.51 (0.13)	0.39 (0.10)
**mesh18**	438898	2192445	0.74 (0.11)	1.07 (0.15)	9051204	45692821	0.51 (0.13)	0.39 (0.10)
**mesh19**	536860	2663864	0.74 (0.11)	1.06 (0.15)	10991686	55387888	0.51 (0.13)	0.39 (0.10)
**mesh20**	556012	2712110	0.73 (0.11)	1.06 (0.15)	11252728	56409760	0.51 (0.13)	0.39 (0.10)
**mesh21**	511855	2544717	0.74 (0.11)	1.07 (0.15)	10522190	53041672	0.51 (0.13)	0.39 (0.10)
**mesh22**	537441	2679368	0.74 (0.11)	1.06 (0.15)	11035397	55662824	0.51 (0.13)	0.39 (0.10)
**mesh23**	407578	1991085	0.73 (0.11)	1.06 (0.15)	8266657	41491489	0.51 (0.13)	0.39 (0.10)
**mesh24**	462282	2257529	0.73 (0.11)	1.06 (0.15)	9383315	47092793	0.51 (0.13)	0.39 (0.10)

**Fig 6:**
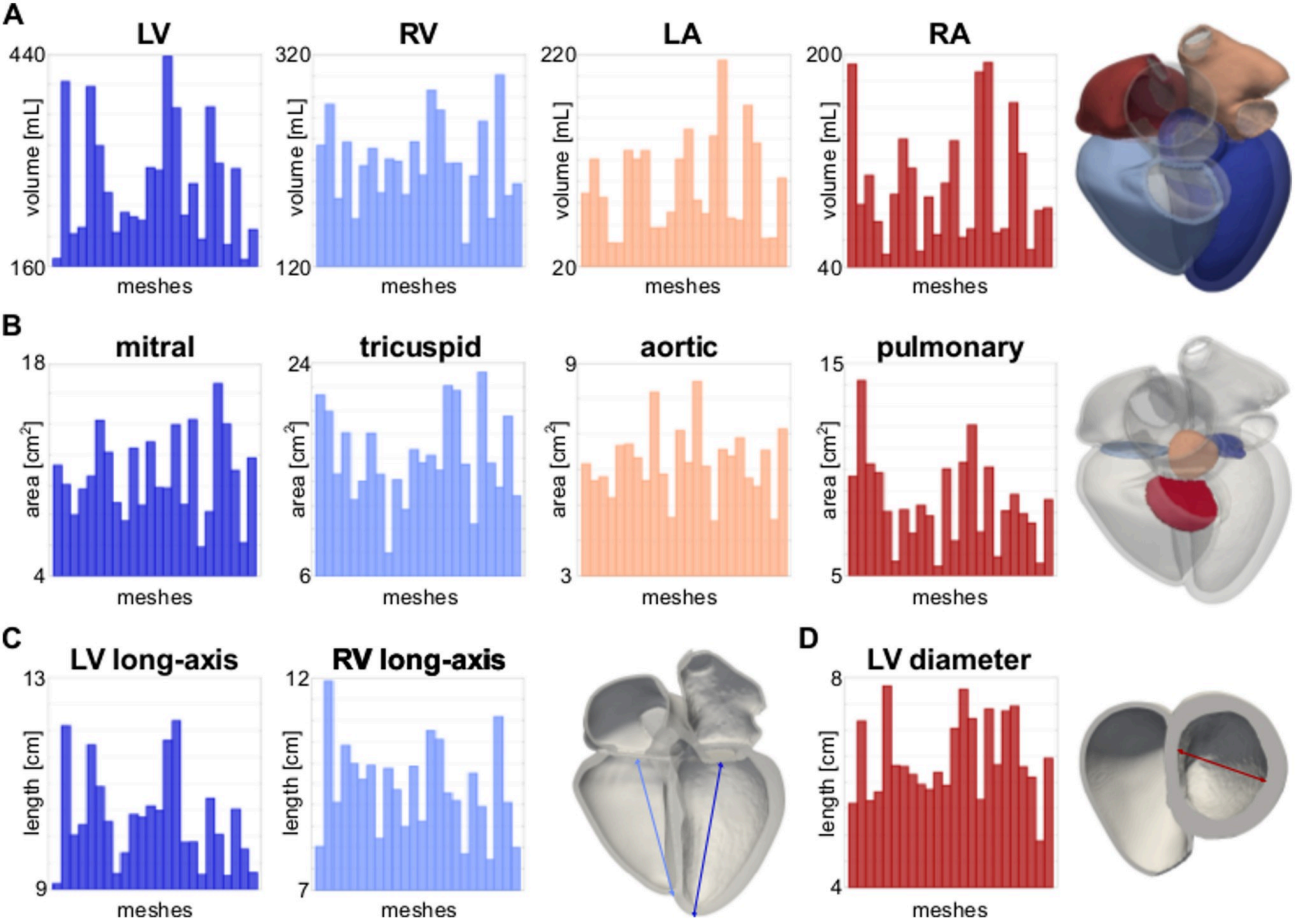
Anatomical differences. **A** LV, RV, LA and RA volumes. **B** Area of the mitral, tricuspid, aortic and pulmonary valve planes. **C** LV and RV long-axis lengths. **D** LV diameter. All quantities are shown for the twenty-four meshes.

We quantified anatomical differences within the cohort. Fig 6A shows the volume of the LV, RV, LA and RA for the twenty-four meshes. Fig 6B shows the area of the mitral, tricuspid, aortic and pulmonary valve planes. Fig 6C shows the LV and RV long-axis length. The LV long-axis length was computed as the distance between the LV most apical point on the epicardium and the center of mass of the mitral valve plane. The RV long-axis was computed by projecting the LV most apical point onto the RV-LV junction intersected with the RV epicardium and by computing its distance from the center of mass of the tricuspid valve plane.

Fig 6D shows the LV diameter, computed using the UVC. We defined a plane below the aortic outflow tract by thresholding the apico-basal (or longitudinal) coordinate between 0.78 and 0.82. We chose these values as this approximates the cutting plane below the LV and RV outflow tracts for all the meshes. Then, we took two points on the LV endocardium representing the opposite ends of the chamber. The point on the LV free wall was taken as the point with the rotational coordinate closest to *π* in UVCs. The point on the side of the septum was taken as the point with the rotational coordinate closest to 0 in UVCs. The LV diameter was computed as the distance between these two points. In addition, we show the variability of the LV wall thickness, and local curvature of the endocardial surface of the LV, RV, LA and RV, which are important quantities for the Laplace law and wall stress estimation in S1 Appendix.

### Electro-mechanics simulations

**Fig 7:**
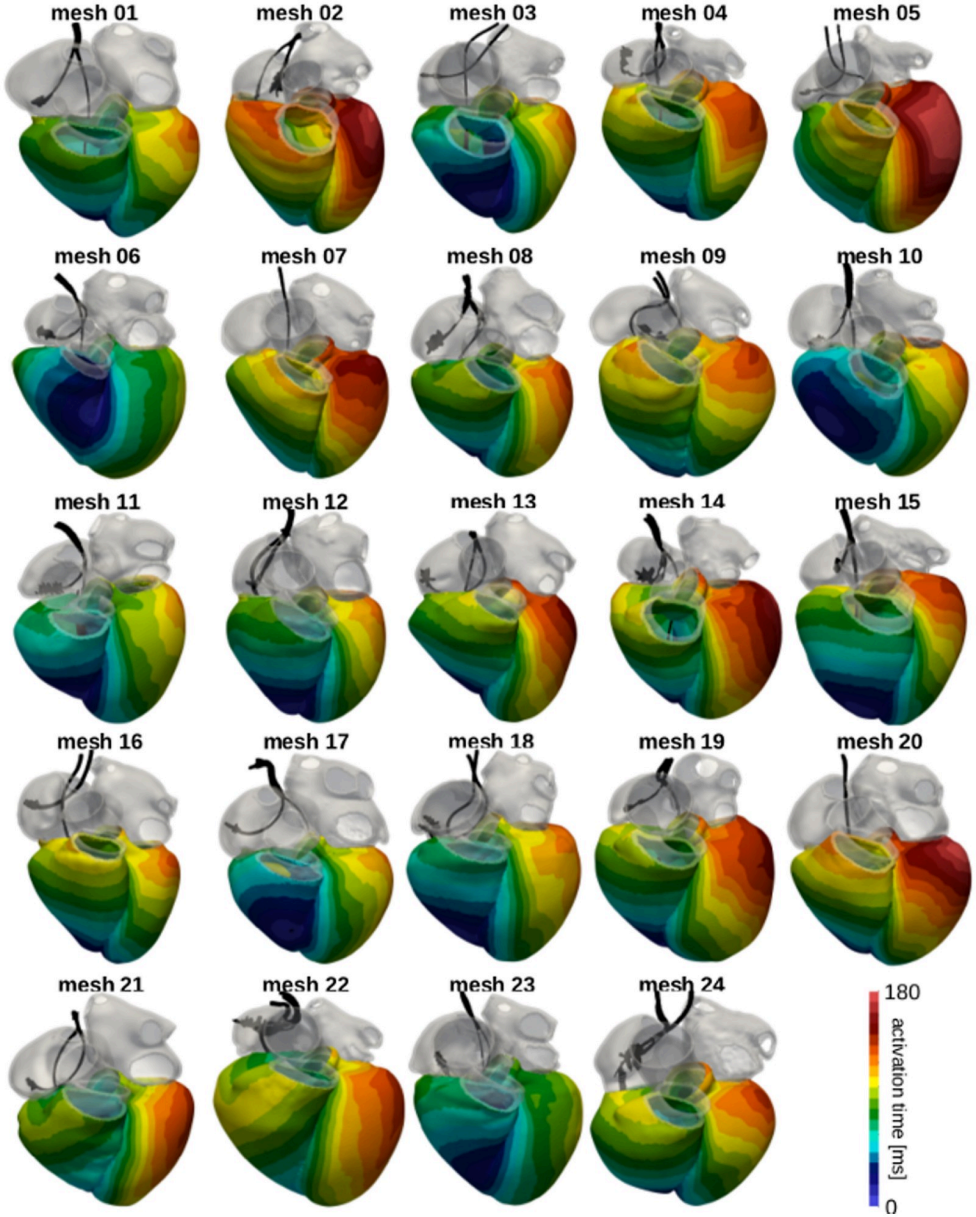
Simulated activation times. The images show an anterior view of all meshes with the ventricles coloured according to the local activation time computed by the eikonal model. The grey regions were excluded by the eikonal solve and were therefore passive. The black structures represent the CRT leads segmented from the CT images. The RV lead was used as the initial activation point.

Results for the electro-mechanics simulation tests are summarised in Table 4. Fig 7 shows ventricular activation times resulting from the eikonal model solved on the twenty-four meshes. The black structures represent the CRT leads segmented from the ED CT images. The RV lead was used to define the initial activation site. We also show changes in transmembrane potential V_m_ (top row) and active tension (bottom row) over time on mesh 01 in Fig 5B.

**Table 4:** Electro-mechanics test simulations. The table summarises results for the electro-mechanics simulation tests. For each mesh, we report the LV and RV latest activation times (LAT), the ejection fraction (EF) and the stroke volume (SV). The last row reports the mean ± the standard deviation of these values.

	LV LAT [ms]	RV LAT [ms]	LV EF [%]	RV EF [%]	LV SV [mL]	RV SV [mL]
**mesh01**	133	101	37	30	62	71
**mesh02**	174	137	37	25	149	68
**mesh03**	154	105	37	31	75	58
**mesh04**	142	121	37	31	49	73
**mesh05**	183	120	36	30	143	50
**mesh06**	138	115	35	32	110	69
**mesh07**	156	118	36	28	93	64
**mesh08**	140	100	36	31	73	60
**mesh09**	141	120	35	28	81	62
**mesh10**	160	103	34	31	78	68
**mesh11**	132	92	36	32	79	60
**mesh12**	151	118	34	29	99	69
**mesh13**	159	106	35	30	99	62
**mesh14**	174	131	36	28	157	82
**mesh15**	167	113	35	29	130	79
**mesh16**	131	112	35	28	81	61
**mesh17**	144	124	35	29	95	64
**mesh18**	136	83	36	32	71	45
**mesh19**	156	111	34	30	125	62
**mesh20**	156	110	34	30	102	77
**mesh21**	141	98	33	30	63	50
**mesh22**	144	109	35	32	100	96
**mesh23**	116	108	36	30	61	55
**mesh24**	141	108	36	32	74	64
	149±16	111±12	35±1	30±2	95±28	65±11

**Fig 8:**
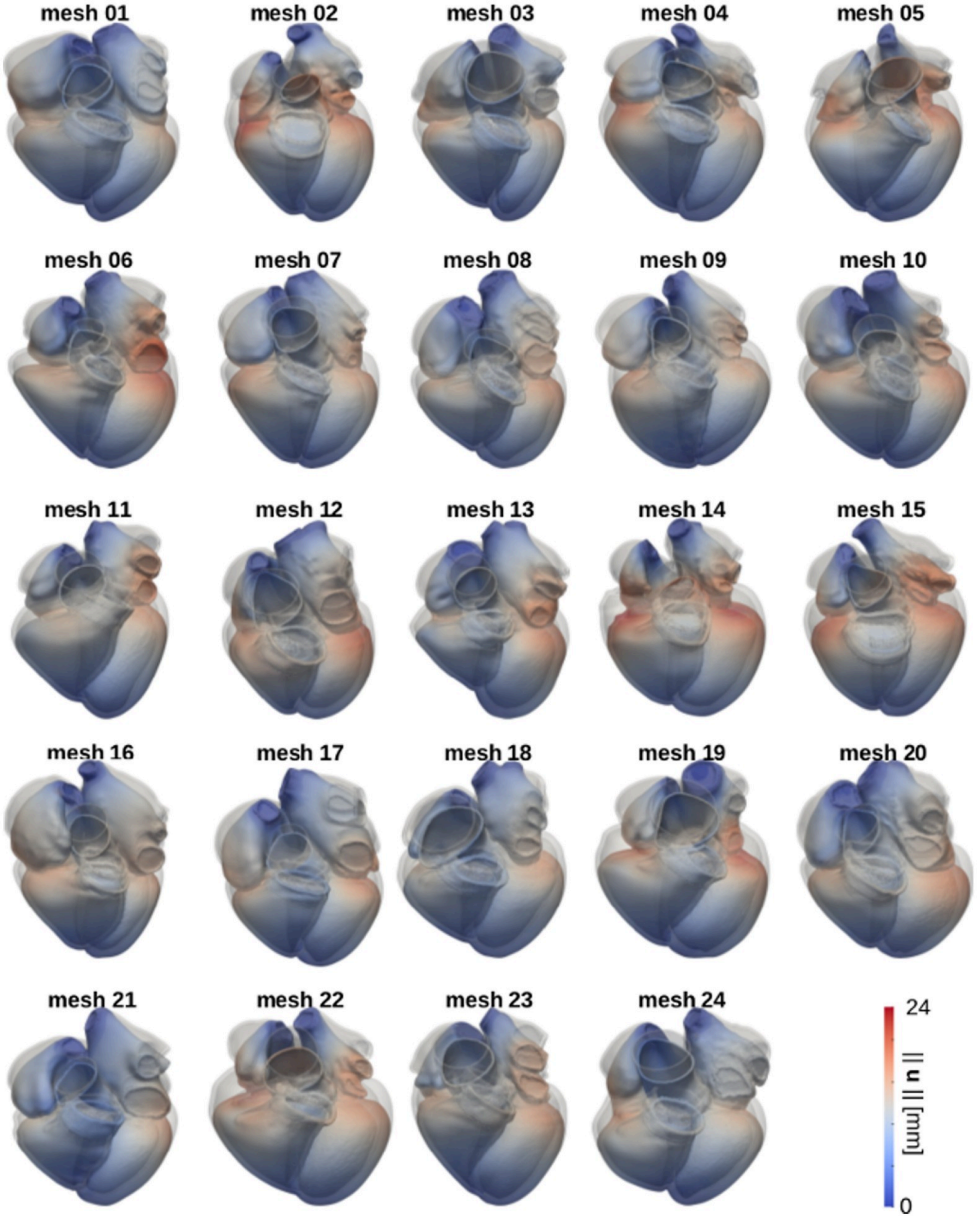
Simulated free active contraction. The images show the results for the free contraction simulations run on all meshes. For each mesh, we show the configuration at the end of LV systole (coloured according to the magnitude of the displacement vector ||**u**|| in mm) and the initial configuration (grey geometry).

Fig 8 shows the results for the free mechanical contraction simulations on the twenty-four meshes. The three-dimensional geometries represent the end-systolic state (coloured according to the displacement magnitude) and the ED state (grey). S7 Fig in the Supporting Information shows that elements undergo significant distortion, leading to an overall decrease of element quality during ventricular contraction. Nevertheless, free contraction simulation tests ran for a whole cardiac cycle on all twenty-four meshes. We therefore concluded that the starting element quality of all meshes was good enough for the elements to endure deformation due to active contraction.

We ran additional simulations with healthy passive stiffness in cases with LV EDV within the plausible physiological healthy range to demonstrate that we could achieve a healthy LV EF. These simulations resulted in an average EF of 50% (range: 48%-53%) and 44% (range 42%-46%) for the LV and the RV, respectively. This shows that the element quality of our meshes is high enough to endure an active contraction with LV EF in healthy ranges when LV EDV falls within normal values.

## Discussion

We generated the first publicly available cohort of twenty-four four-chamber meshes from HF patients with our semi-automatic pipeline. The elements of the resulting geometries have high quality. We showed that our meshes are suitable for electro-mechanical simulations by successfully running test simulations for electrical activation and free mechanical contraction. We proved that our meshes can be refined to a desired edge length, making our cohort potentially suitable for more complex electrophysiology problems. Both coarse (1.1mm average edge length) and fine (0.39mm average edge length) versions of all twenty-four meshes are publicly available to download, in order to promote the development of computational cardiac electro-mechanics.

### Mesh characteristics and usability

Solver convergence in electrophysiology simulations is highly dependent on mesh resolution depending on the model used to simulate electrical activation (for example eikonal, monodomain and bidomain). The average edge length of our meshes was close to the target edge length of 1mm, see Table 3. This element size makes the meshes suitable for electrical activation simulations with the eikonal model [42], and at the same time significantly reduces the computational costs of the simulations. For a more complex electrical activation model (e.g. monodomain or bidomain formulations), a fine mesh resolution is required [42, 62, 65]. We showed that our meshes can be resampled to a desired edge length by generating a finer version of all twenty-four meshes (average edge length 0.39mm) with a publicly available tool. The user can potentially refine our meshes to an even smaller edge length if desired.

As opposed to electrophysiology simulation, in which the most important mesh characteristic for solver convergence is mesh resolution while element distortion does not represent an issue, solver convergence in mechanics simulations is affected by (but not only) element quality. Mesh quality was identified in the past as a metric to determine the stability of the mechanics simulation for different types of elements [53, 64, 66]. The threshold on element distortion is, however, sensitive to the type of elements of the mesh. For cubic-Hermite elements, Lamata et al defined a scaled Jacobian of 0.33 to determine *a-priori* stability of cardiac mechanics simulations for a given mesh [66, 67]. The use of cubic-Hermite elements can reduce the degrees of freedom of the mesh. However, reduced computational costs gained by a lower number of degrees of freedom comes at the cost of losing accuracy in representing cardiac anatomical structures (e.g. valve planes, LV and RV outflow tract, atria) and an increase in mesh generation complexity [66, 68]. Tetrahedral elements offer a detailed representation of the whole heart. The elements of our meshes had an average scaled Jacobian higher than 0.7. Although we did not test the effect of lower element quality on simulation stability, the quality of our meshes was high enough to run free active contraction successfully on all twenty-four meshes. S7 Fig in the supporting information shows mesh quality of mesh 03 changing over time for a free contraction simulation. Our results show that mesh quality decreases during the simulation due to large deformations. For this reason, it is important for the initial mesh to have high quality elements so that element deformation does not lead to degenerated elements.

### Cohort anatomical variability

The size of the heart is used as a determinant of cardiac function, and is identified as an independent predictor of cardiac events in patients with cardiovascular diseases [69–71]. LV ED volumes in our cohort (269±78mL) are consistent with values reported for HF patients in CRT clinical trials (REVERSE: 269±93mL [72]; STARTER: 189±70mL [73]; MIRACLE-ICD: 317±98mL [74]). Despite high variations in both our cohort LV volumes and literature values, these were significantly higher than values reported in the literature for healthy controls (136±21mL [75]). The same observations apply to values for LV diameter. Our cohort had a LV diameter of 64±8mm, similar to values reported in CRT clinical trials (MIRACLE: 70±10mm [76]; REVERSE: 69±9mm [72]; MUSTIC: 71±9mm [77]; MIRACLE-ICD: 76±10mm [74]). As for the LV ED volume, LV diameter values were larger than values reported in the literature for healthy controls (50±8mm [78]). Gibson et al [79] reported a linear correlation of 74% between LV ED volume and LV diameter. We measured a similar correlation of 81% within our cohort. RV, LA and RA size measurements in HF patients are more scarse in the literature compared to LV size measurements. RV ED volume for our cohort (219±39mL) was dilated compared to values reported in the literature for healthy controls (136±24mL [80]). LA volume in our cohort (98±45mL) was also increased compared to values reported for healthy individuals (males: 32±11mL, females: 27±10mL [81]). On the other hand, RA volume in our cohort (102±45mL) falls in ranges for healthy controls (100±20mL [82]). Cardiac chamber volumes indicate that patients in our cohort underwent LV, RV and LA remodelling. Ventricular remodelling is known to happen in HF patients [83]. Ventricular failure in turn causes LA overload, with LA increased pressure and volume [84].

### Limitations

Our meshes do not account for intricate and complex microstructures of ventricular endocardium. Highly detailed models of the rabbit [85–88], dog [88] and human [88–90] ventricles were generated in the past. These anatomies were based on *ex-vivo* imaging data, as *in-vivo* imaging techniques still do not allow for an enough high resolution to visualise such fine structures. Our four-chamber geometries were generated from *in-vivo* human CT clinical datasets. Therefore we were not able to include endocardial structures in our ventricular geometries. Ventricular trabeculations were reported to affect ventricular haemodynamics [91, 92]. Ventricular endocardial structures were however found to have a marginal effects on strains [93] and arrhythmias re-entry [94]. Therefore our meshes might not be suitable for detailed haemodynamics simulations, but can be used for mechanics and electrophysiology simulations (when refined if required by the activation model).

Our ventricular models also do not include a Purkinje network. The Purkinje tree plays an important role in ventricular fibrillation [95, 96] and arrhythmias [97–101]. From a modelling point of view, including the ventricular fast conduction system is necessary to simulate physiological ECGs [89]. Purkinje tree morphology was also reported to affect ECG shape [102]. Therefore, if the user intends to use our meshes to investigate ventricular arrhythimas or fibrillation, or to simulate ECG resulting from activation, the Purkinje network can be included using publicly available tools (https://github.com/fsahli/fractal-tree) or by mapping a pre-existing tree using the UVCs [41], also provided for each mesh.

Our geometries do not offer a detailed model of atrial structures. The LAA was not included in the geometries because the CT volume often did not cover it. Other structures such as crista terminalis, pectinate muscles and fossa ovalis were not identified as separate tags in the atrial meshes. However, these can be included based on previous studies offering more detailed representation of LA and RA anatomical regions [5, 103].

Another limitation of our atrial model is that we did not include atrial fibres. Atrial fibers are more challenging to embed in three-dimensional geometries compared to ventricular fibres, due to complex atrial anatomy and scarcity of data about fibre orientation in atrial myocardium. It is however possible for the user to define atrial fibres using a rule-based method [103, 104] or by mapping a pre-existing fibre field using the universal atrial coordinated (UACs) [105–107].

The aim of our study was not to provide a fully detailed model of the heart. We instead provided a reference set of twenty-four four-chamber anatomies which external users can add features (such as Purkinje network, atrial regions, atrial fibres etc…) to. We believe that our database constitutes a significant step forward for the computational modelling community, as it provides the first publicly available database of four-chamber heart models.

## Conclusion

We built a cohort of twenty-four four-chamber heart meshes from HF patients CT data. The elements of our meshes have high quality, making them suitable for electro-mechanics simulations. We verified this by running electro-mechanics simulation tests for ventricular electrical activation and free contraction. The twenty-four geometries can be refined and integrated with more purpose-specific features, and used for large cohort computational studies and as a data set for verification of numerical methods for cardiac electro-mechanics.

## Supporting information

S1 Fig Segmentation cases 01 to 04. The image shows the segmentation for the components included in the final tetrahedral mesh for cases 01 to 04 overlapped with the end-diastolic CT image. For each case, we show a four-chamber axial view, two sagittal and two coronal views. Abbreviations: left ventricle (LV), right ventricle (RV), left atrium (LA), right atrium (RA), aorta (Ao) and pulmonary artery (PA).
(PDF)

S2 Fig Segmentation cases 05 to 08. The image shows the segmentation for the components included in the final tetrahedral mesh for cases 05 to 08 overlapped with the end-diastolic CT image. For each case, we show a four-chamber axial view, two sagittal and two coronal views. Abbreviations: left ventricle (LV), right ventricle (RV), left atrium (LA), right atrium (RA), aorta (Ao) and pulmonary artery (PA).
(PDF)

S3 Fig Segmentation cases 09 to 12. The image shows the segmentation for the components included in the final tetrahedral mesh for cases 09 to 12 overlapped with the end-diastolic CT image. For each case, we show a four-chamber axial view, two sagittal and two coronal views. Abbreviations: left ventricle (LV), right ventricle (RV), left atrium (LA), right atrium (RA), aorta (Ao) and pulmonary artery (PA).
(PDF)

S4 Fig Segmentation cases 13 to 16. The image shows the segmentation for the components included in the final tetrahedral mesh for cases 13 to 16 overlapped with the end-diastolic CT image. For each case, we show a four-chamber axial view, two sagittal and two coronal views. Abbreviations: left ventricle (LV), right ventricle (RV), left atrium (LA), right atrium (RA), aorta (Ao) and pulmonary artery (PA).
(PDF)

S5 Fig Segmentation cases 17 to 20. The image shows the segmentation for the components included in the final tetrahedral mesh for cases 17 to 20 overlapped with the end-diastolic CT image. For each case, we show a four-chamber axial view, two sagittal and two coronal views. Abbreviations: left ventricle (LV), right ventricle (RV), left atrium (LA), right atrium (RA), aorta (Ao) and pulmonary artery (PA).
(PDF)

S6 Fig Segmentation cases 21 to 24. The image shows the segmentation for the components included in the final tetrahedral mesh for cases 21 to 24 overlapped with the end-diastolic CT image. For each case, we show a four-chamber axial view, two sagittal and two coronal views. Abbreviations: left ventricle (LV), right ventricle (RV), left atrium (LA), right atrium (RA), aorta (Ao) and pulmonary artery (PA).
(PDF)

S7 Fig Element quality change during a mechanics simulation. **A** The plot shows error bars (mean ± standard deviation) of the mesh quality over time for a free contraction simulation for mesh 03. The other plots show mesh quality variations over simulation time for different regions of the mesh: **B** LV, **C** RV, **D** LA and **E** RA. Average mesh quality decreases from 0.74 to a minimum of 0.63, reached at maximum ventricular contraction. Change in mesh quality is driven by the LV and the RV, for which the average quality decreases of 16% and 20% from ED to end-systole, respectively. LA and the RA mesh quality, which are not actively contracting in the simulation and are therefore passive, underwent small variations (4% and 3%, respectively). The same applied to all the other labels of the mesh (results not shown).
(PDF)

S1 Appendix LV myocardium thickness and endocardial curvature variability. (PDF)

S2 Appendix Penalty map for spring stiffness. (PDF)
