# Supplementary figures and images for: A publicly available virtual cohort of four-chamber heart meshes for cardiac electro-mechanics simulations
Source: PLoS One. 2020 Jun 26;15(6):e0235145. doi: 10.1371/journal.pone.0235145 (PMC7319311; doi:10.1371/journal.pone.0235145)

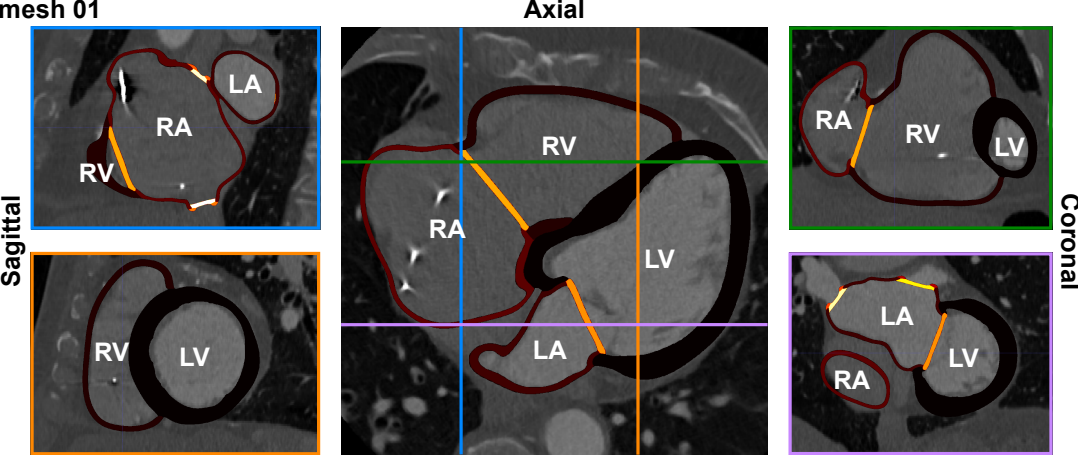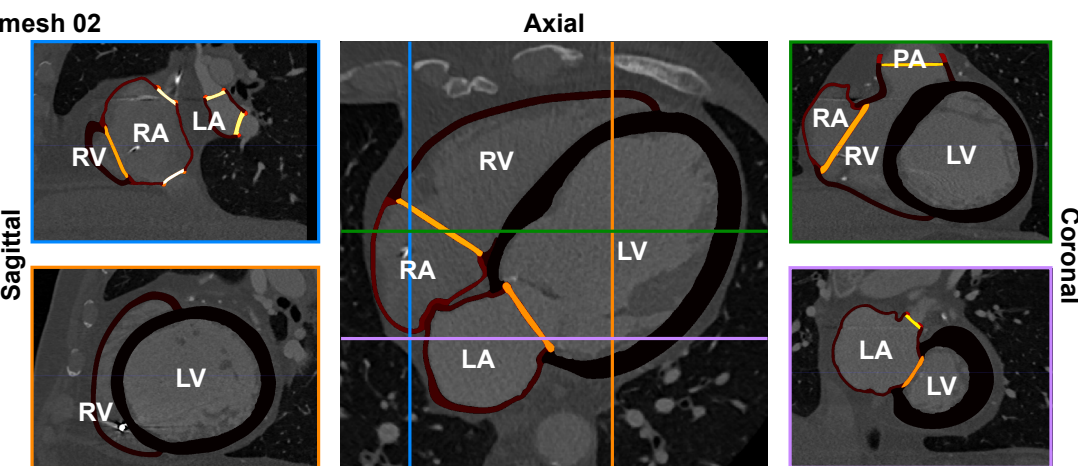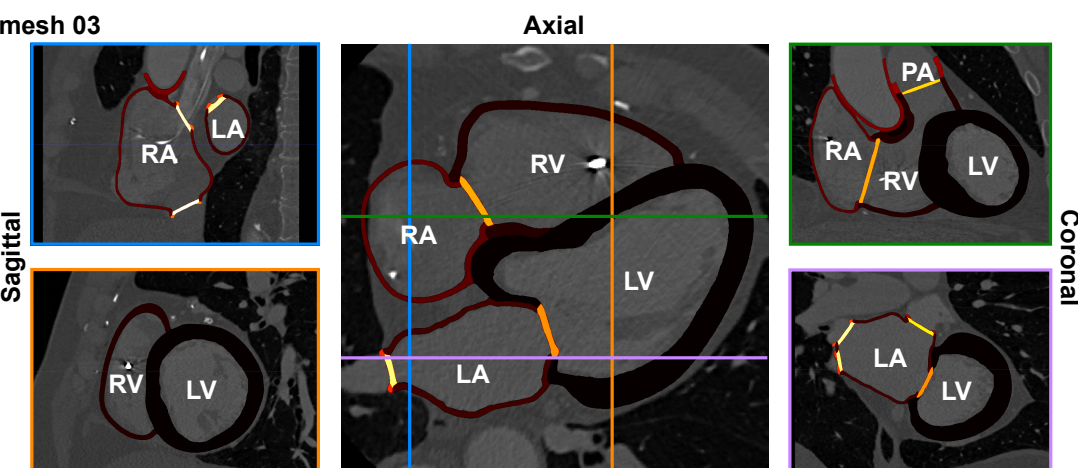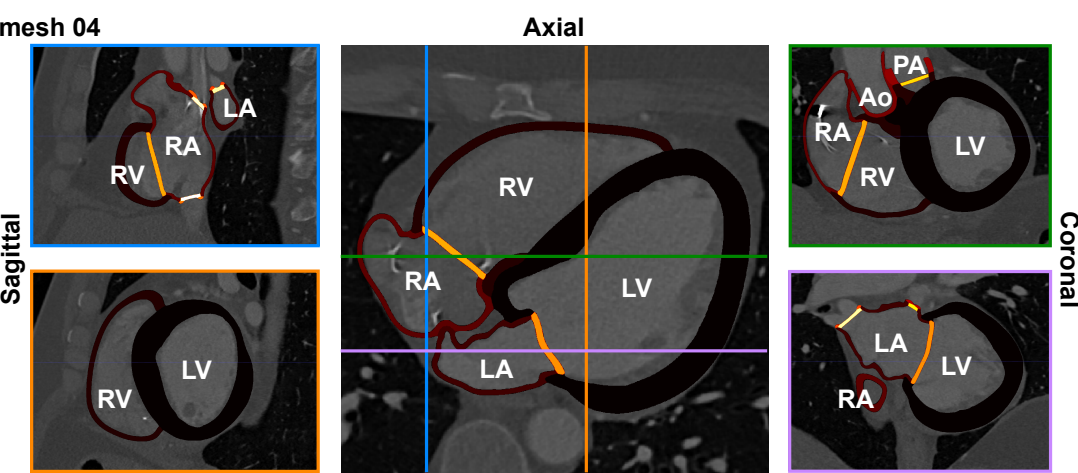

Supplement: S1 Fig — The image shows the segmentation for the components included in the final tetrahedral mesh for cases 01 to 04 overlapped with the end-diastolic CT image. For each case, we show a four-chamber axial view, two sagittal and two coronal views. Abbreviations: left ventricle (LV), right ventricle (RV), left atrium (LA), right atrium (RA), aorta (Ao) and pulmonary artery (PA). (PDF) [file pone.0235145.s001.pdf]

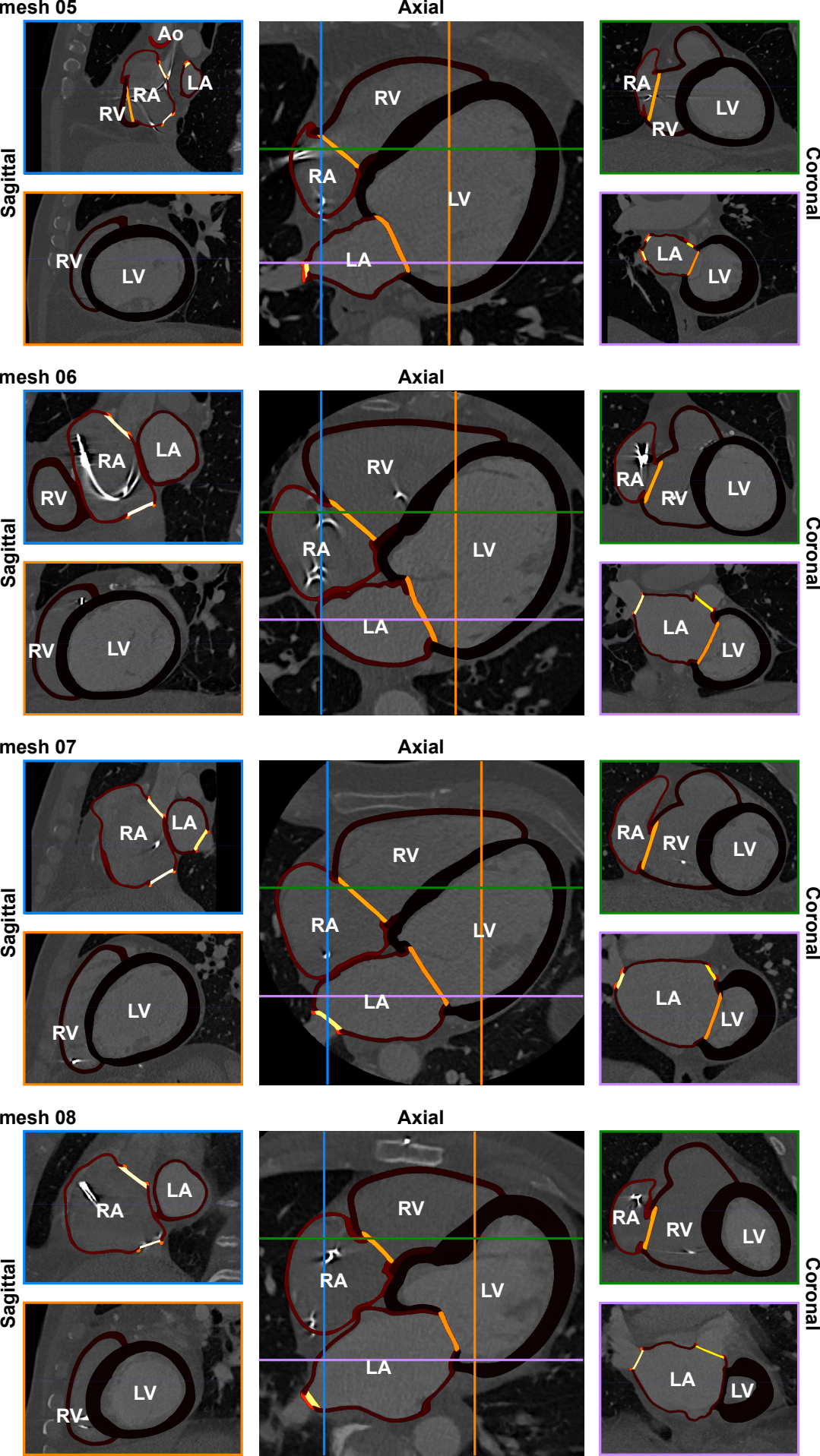

Supplement: S2 Fig — The image shows the segmentation for the components included in the final tetrahedral mesh for cases 05 to 08 overlapped with the end-diastolic CT image. For each case, we show a four-chamber axial view, two sagittal and two coronal views. Abbreviations: left ventricle (LV), right ventricle (RV), left atrium (LA), right atrium (RA), aorta (Ao) and pulmonary artery (PA). (PDF) [file pone.0235145.s002.pdf]

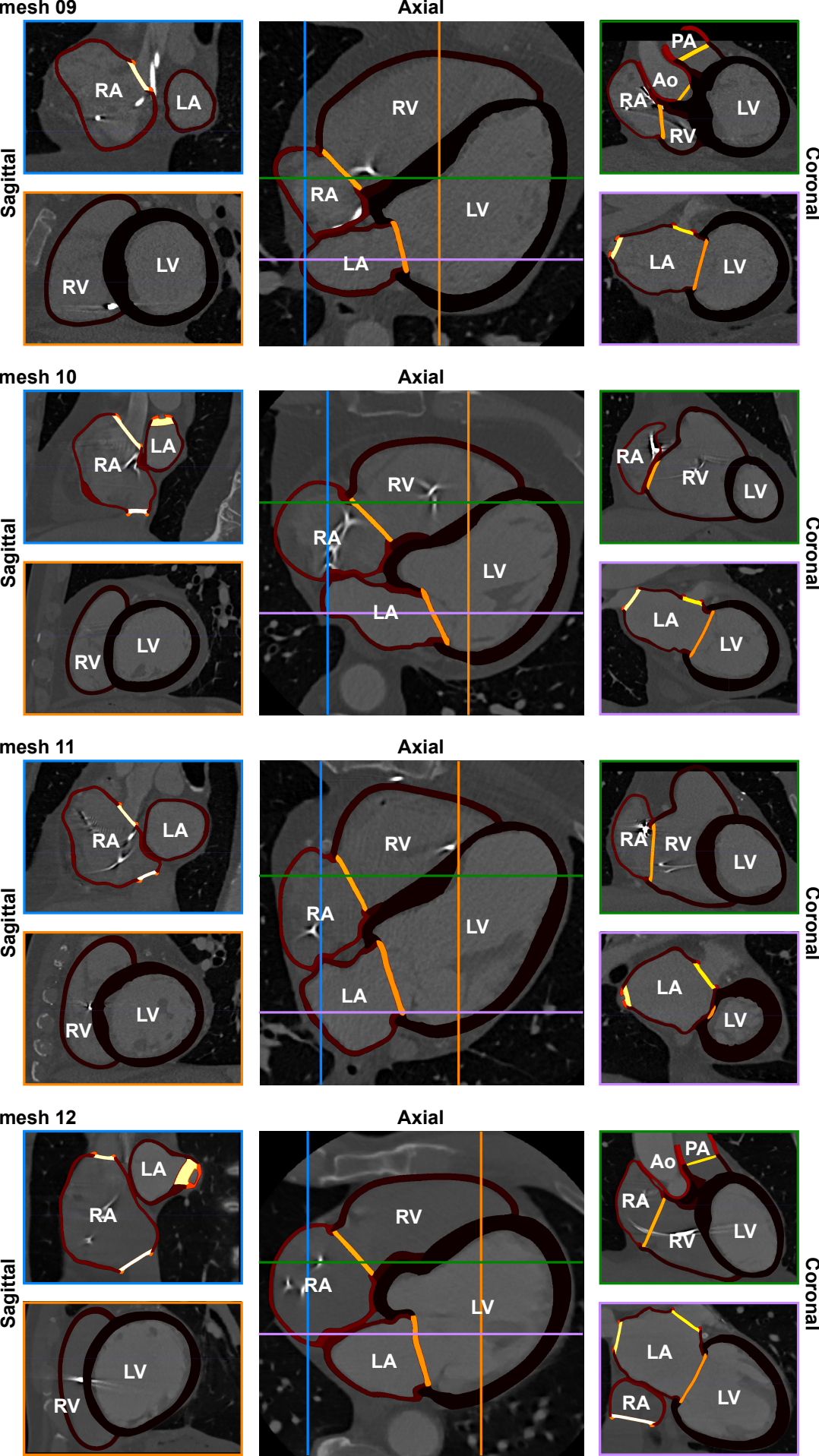

Supplement: S3 Fig — The image shows the segmentation for the components included in the final tetrahedral mesh for cases 09 to 12 overlapped with the end-diastolic CT image. For each case, we show a four-chamber axial view, two sagittal and two coronal views. Abbreviations: left ventricle (LV), right ventricle (RV), left atrium (LA), right atrium (RA), aorta (Ao) and pulmonary artery (PA). (PDF) [file pone.0235145.s003.pdf]

## mesh 17

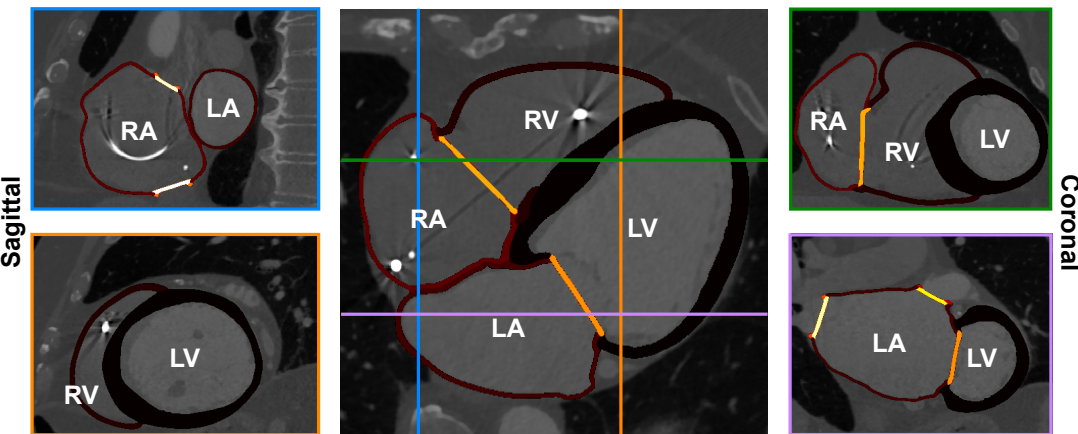

**mesh 18**

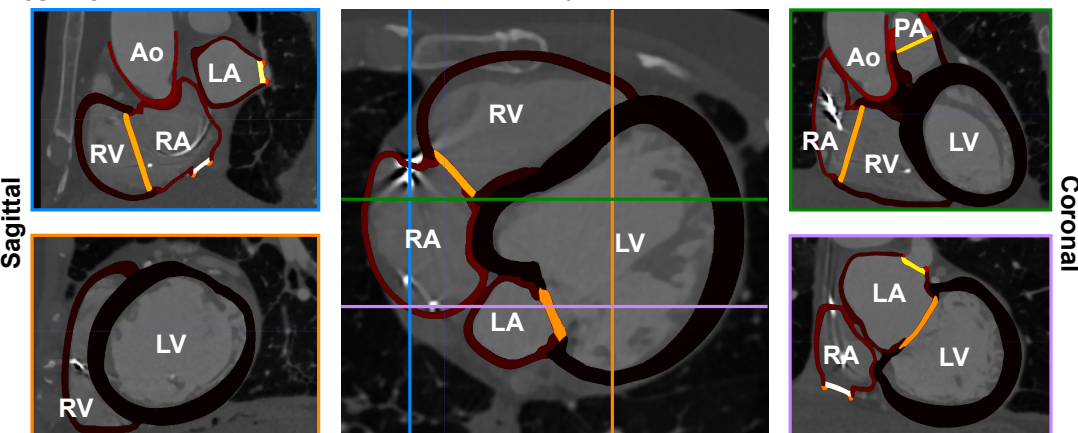

**mesh 19**

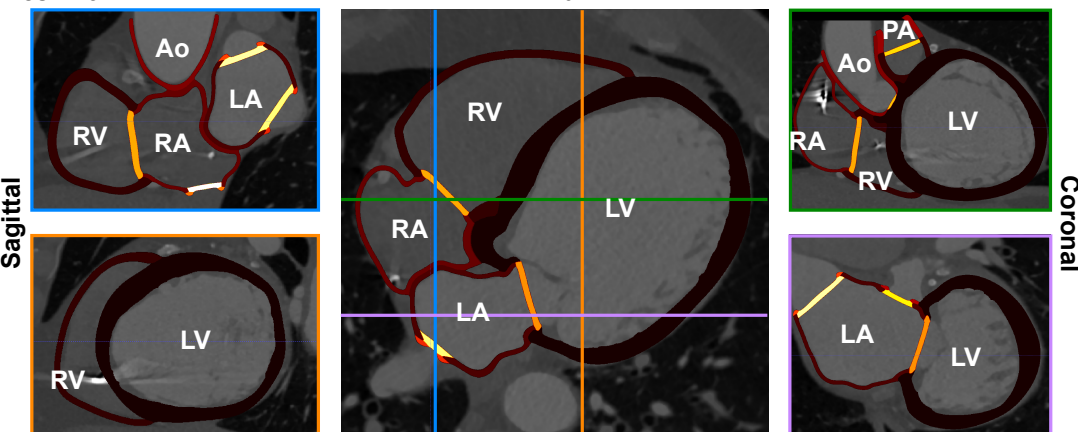

**mesh 20**

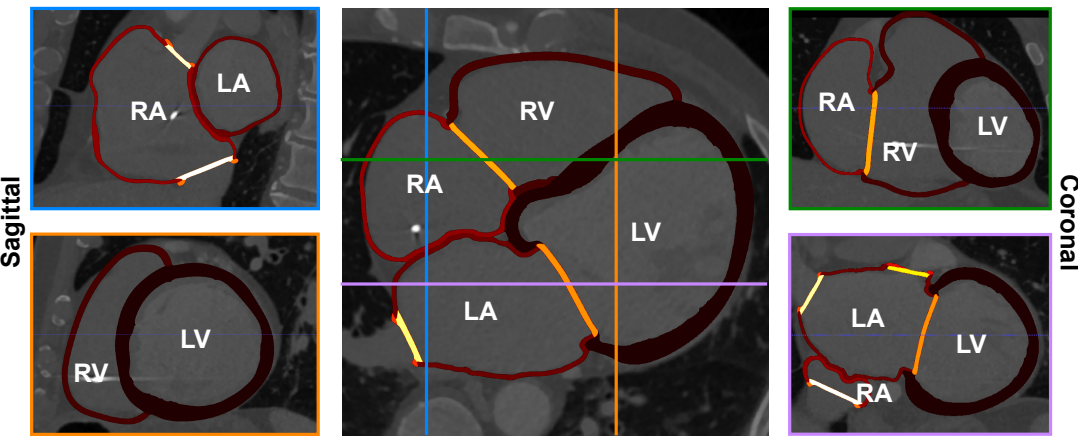

Supplement: S5 Fig — The image shows the segmentation for the components included in the final tetrahedral mesh for cases 17 to 20 overlapped with the end-diastolic CT image. For each case, we show a four-chamber axial view, two sagittal and two coronal views. Abbreviations: left ventricle (LV), right ventricle (RV), left atrium (LA), right atrium (RA), aorta (Ao) and pulmonary artery (PA). (PDF) [file pone.0235145.s005.pdf]

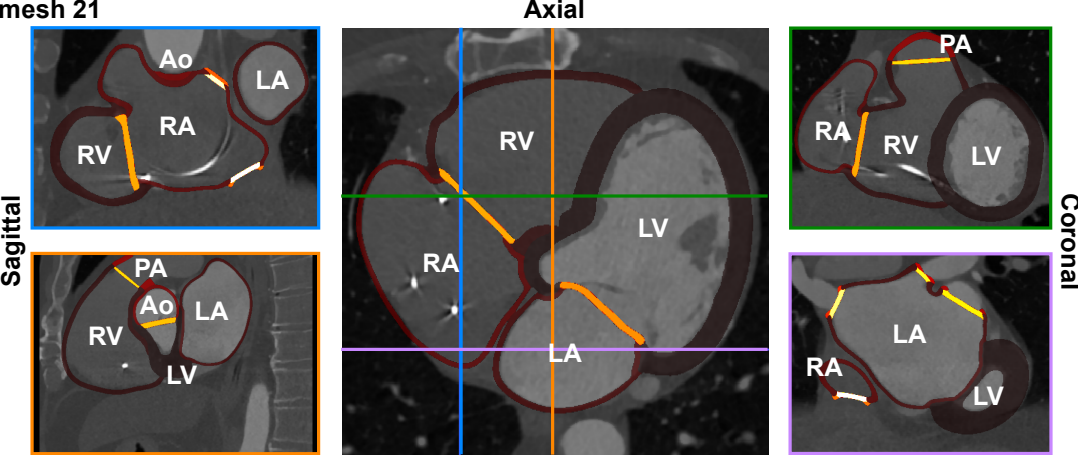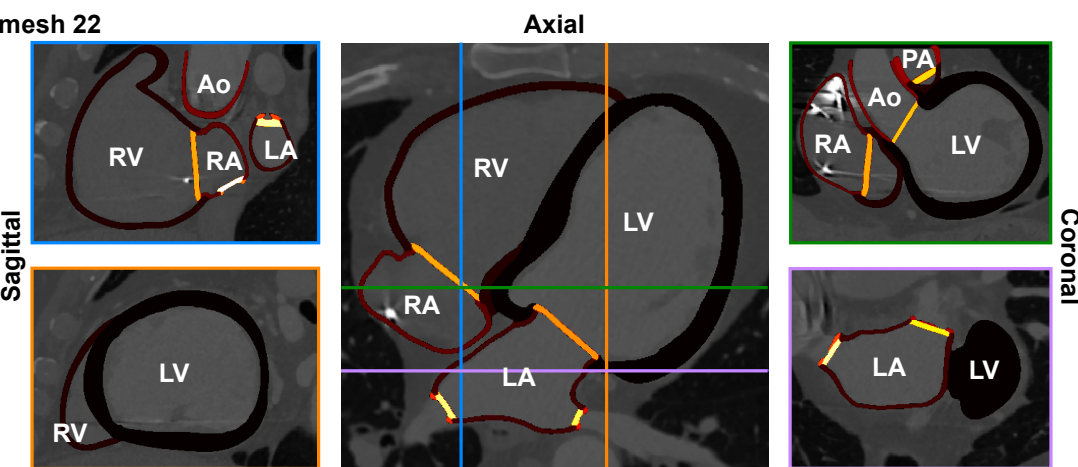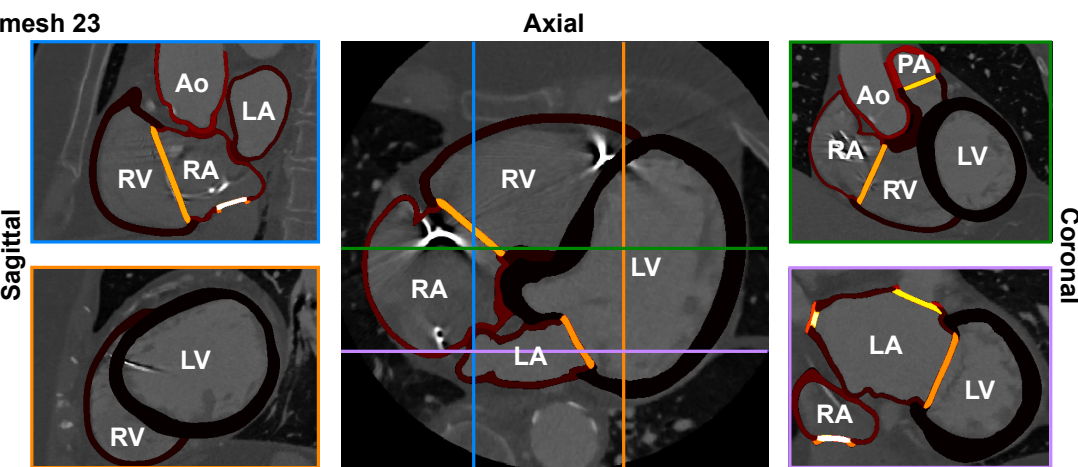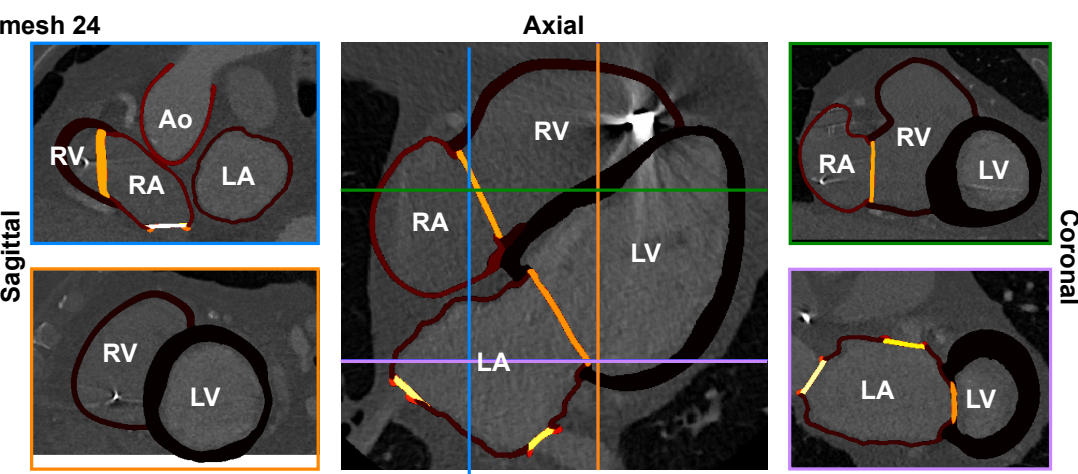

Supplement: S6 Fig — The image shows the segmentation for the components included in the final tetrahedral mesh for cases 21 to 24 overlapped with the end-diastolic CT image. For each case, we show a four-chamber axial view, two sagittal and two coronal views. Abbreviations: left ventricle (LV), right ventricle (RV), left atrium (LA), right atrium (RA), aorta (Ao) and pulmonary artery (PA). (PDF) [file pone.0235145.s006.pdf]

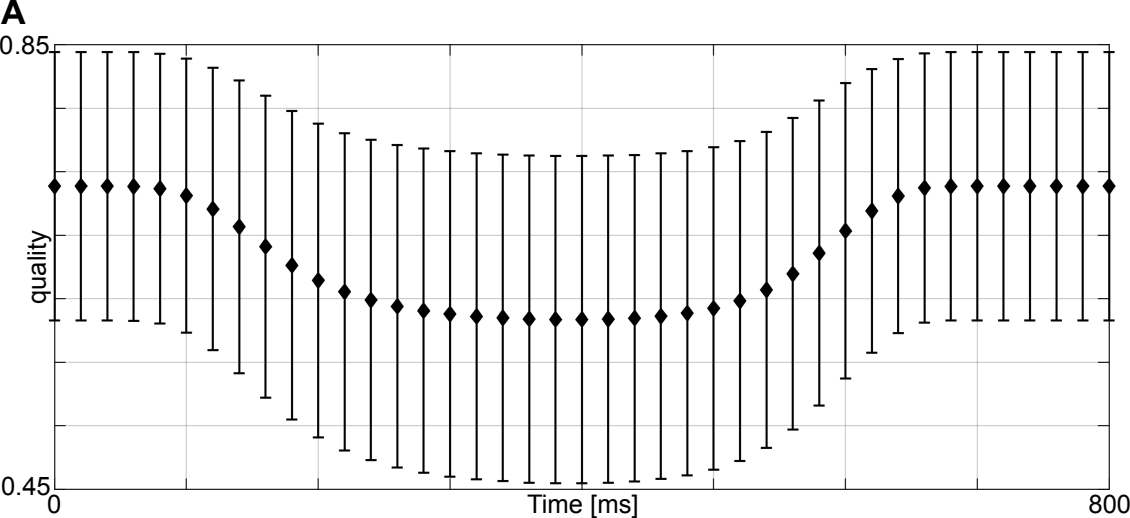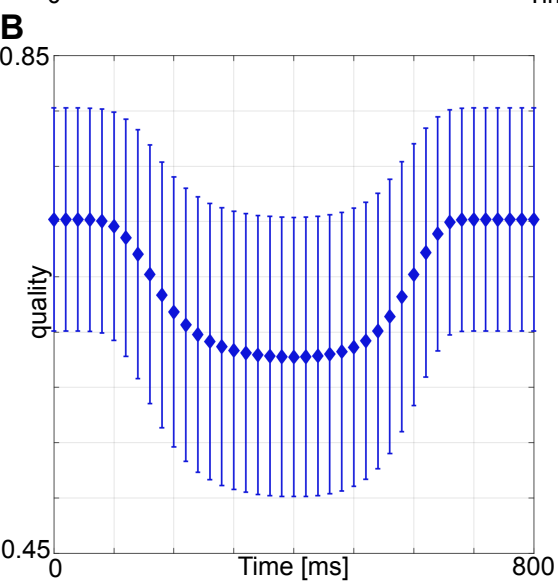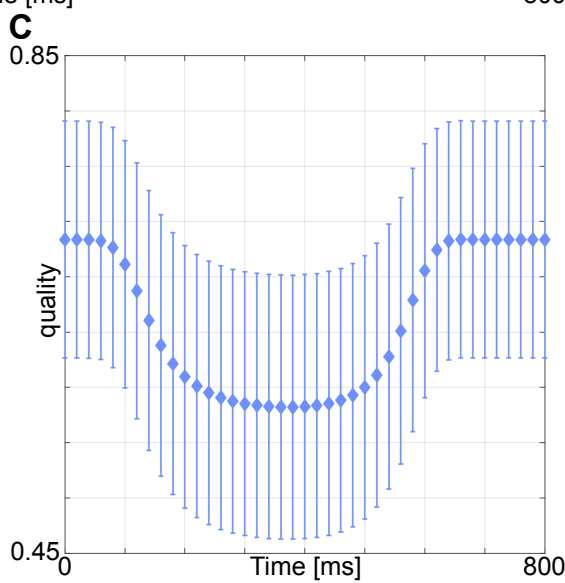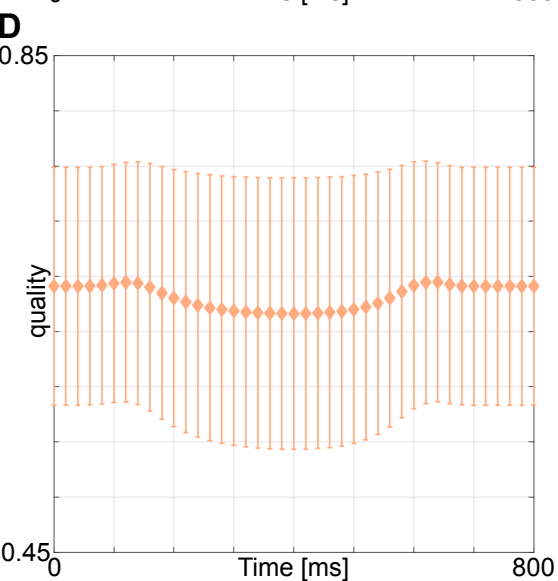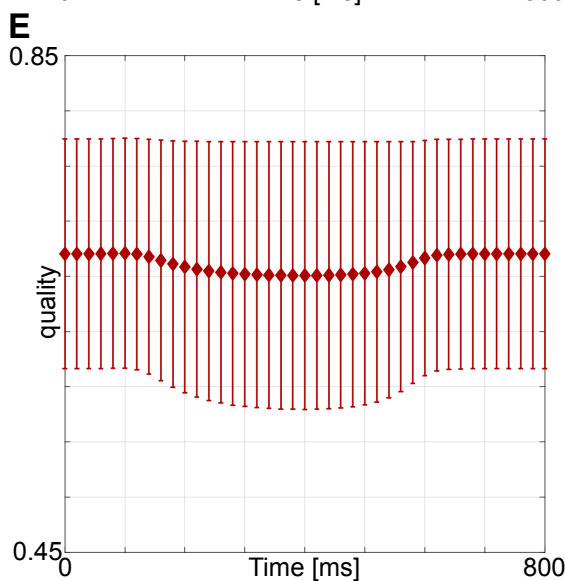

Supplement: S7 Fig — A The plot shows error bars (mean ± standard deviation) of the mesh quality over time for a free contraction simulation for mesh 03. The other plots show mesh quality variations over simulation time for different regions of the mesh: B LV, C RV, D LA and E RA. Average mesh quality decreases from 0.74 to a minimum of 0.63, reached at maximum ventricular contraction. Change in mesh quality is driven by the LV and the RV, for which the average quality decreases of 16% and 20% from ED to end-systole, respectively. LA and the RA mesh quality, which are not actively contracting in the simulation and are therefore passive, underwent small variations (4% and 3%, respectively). The same applied to all the other labels of the mesh (results not shown). (PDF) [file pone.0235145.s007.pdf]
