# Supplementary material for: A publicly available virtual cohort of four-chamber heart meshes for cardiac electro-mechanics simulations
Source: PLoS One. 2020 Jun 26;15(6):e0235145. doi: 10.1371/journal.pone.0235145 (PMC7319311; doi:10.1371/journal.pone.0235145)
Supplement: S4 Fig — The image shows the segmentation for the components included in the final tetrahedral mesh for cases 13 to 16 overlapped with the end-diastolic CT image. For each case, we show a four-chamber axial view, two sagittal and two coronal views. Abbreviations: left ventricle (LV), right ventricle (RV), left atrium (LA), right atrium (RA), aorta (Ao) and pulmonary artery (PA). (PDF) [file pone.0235145.s004.pdf]

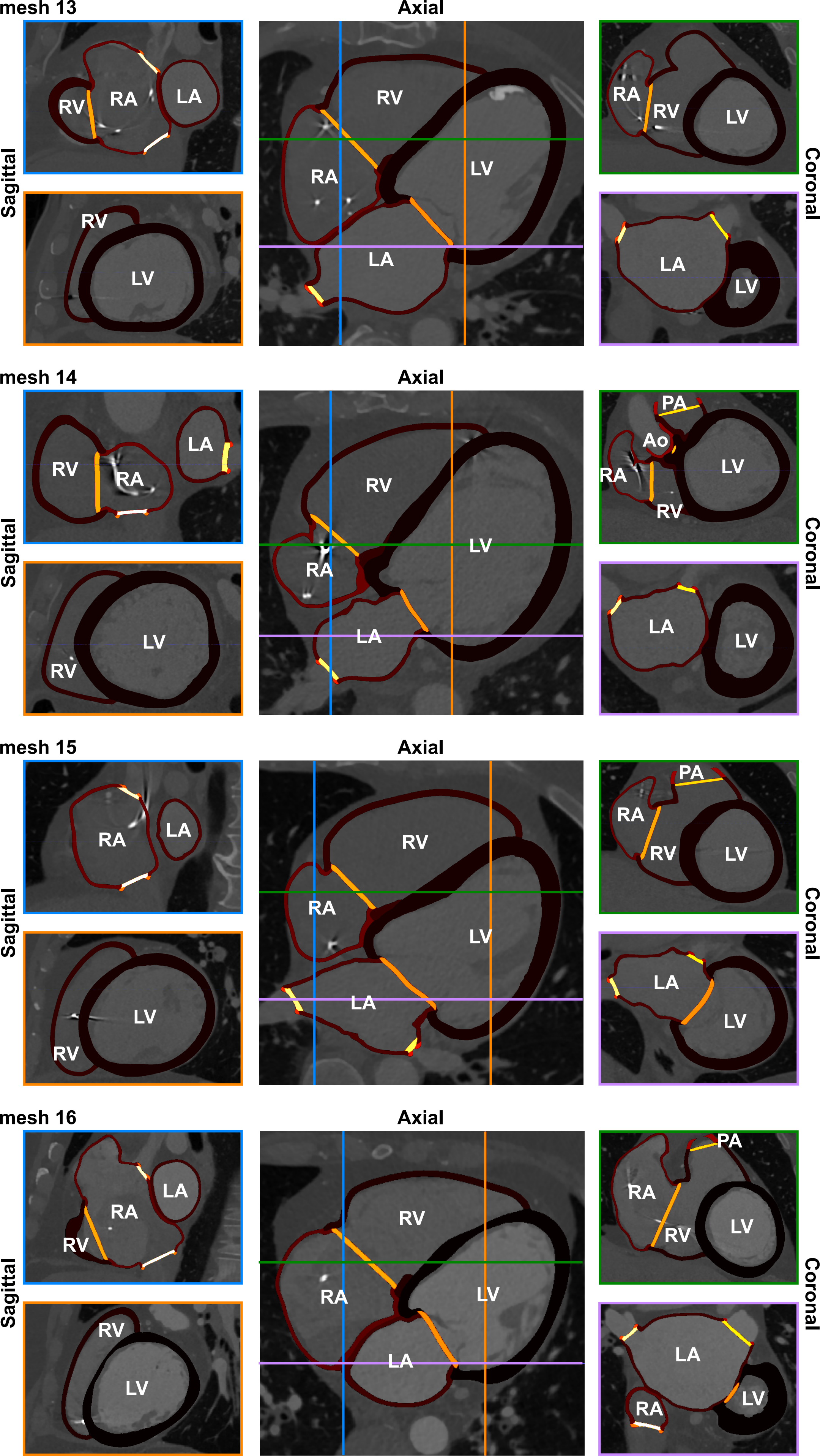
mesh 13
Axial
Sagittal
Coronal
RV
RA
LA
LV
mesh 14
Axial
Sagittal
Coronal
PA
Ao
mesh 15
Axial
Sagittal
Coronal
mesh 16
Axial
Sagittal
Coronal
