# Supplementary material for: A publicly available virtual cohort of four-chamber heart meshes for cardiac electro-mechanics simulations
Source: PLoS One. 2020 Jun 26;15(6):e0235145. doi: 10.1371/journal.pone.0235145 (PMC7319311; doi:10.1371/journal.pone.0235145)
Supplement: S2 Appendix — (PDF) [file pone.0235145.s009.pdf]

Appendix: Penalty Map for Spring Stiffness

In this Appendix, we describe how we derived the map to scale the normal spring stiffness to represent the effect of the pericardium from the image-derived displacement fields. For each patient, we had CT images acquired over 10 or 20 frames over one cardiac cycle. We applied a motion tracking algorithm to assign each voxel with a displacement vector with respect to the initial (end-diastolic) frame [1]. We registered our four-chamber heart mesh to the CT images and we warped it according to the image-derived displacement field. For case 03, motion data were not available.

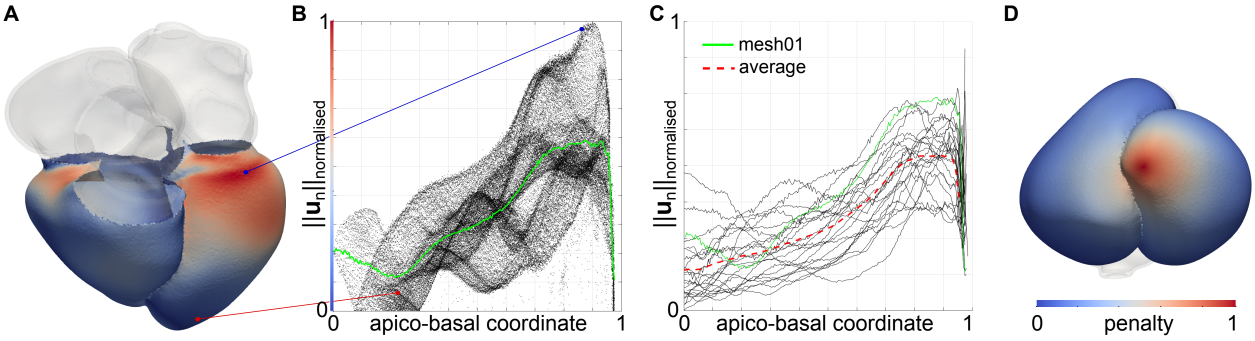

Fig. 1. Scheme for penalty map derivation. A As an example, mesh 01 with the epicardium of the ventricles colored according to the displacement normal to the surface normalised between 0 and 1. **B** Plot of the displacement normal to the surface against the apico-basal coordinate from the universal ventricular coordinates (UVC). 0 represents the apex and 1 represents the base. Each black dot represents a surface triangle of the epicardium. The green line is the average taken over triangles with similar apico-basal coordinate. **C** Average curve for all cases (black lines). The green line represents mesh 01, while the red dashed line represents the average over all the meshes. **D** Resulting scaling penalty map for the stiffness of the normal springs representing the pericardium. The map is shown in an apical view of mesh 01.

Using the warped mesh, we selected the end-systolic configuration as the frame with maximum displacement from ED. We extracted the epicardial surface of the ventricles, and assigned each triangle of the surface with a displacement vector by taking the average displacement vector of the three nodes of the triangle. We projected the end-systolic surface displacement vectors in the normal direction to each triangle. Figure 1A shows the end-systolic epicardial displacement normal to the surface for mesh 01. We normalised the normal displacement between 0 and 1 and we plotted it against the apico-basal coordinate from the UVC. Figure 1B shows such plot for mesh 01, with each of the black points representing one triangle of the epicardial surface. Since we wanted to extract a function from these data, we took an average curve over the triangles with similar apico-basal coordinate values. That gave us an average curve for each case (green curve in Figure 1B and black curves in Figure 1C). We then took an average over all the twenty-three cases (red dashed line in Figure 1C).

From the average displacement curve, we observed that the apex moved the least. Also, triangles with an apico-basal coordinate larger than 0.82 had maximum normal displacement. We therefore assigned these triangles with 0 penalty. To fit the non-linear increase of epicardial normal displacement from apex to base, we fitted a third-degree polynomial function. We finally flipped it to assign the apex with maximum penalty and the base with zero penalty. The final scaling function

we applied is:

$$s(x) = \begin{cases} p_1x^3 + p_2x^2 + p_3x + p4 \ , & x \leq 0.82 \\ 1.0 & x \geq 0.82 \end{cases}$$

where x represents the longitudinal UVC, and p_1 = 1.5266, p_2 = -0.37, p_3 = 0.4964 and p_4 = 0. The penalty map for mesh 01 is shown in Figure 1D. We used this map to scale a spring stiffness of 0.25kPa mm^{-1}. Therefore, the base was not constrained in the normal direction, while the normal displacement at the apex was constrained the most.

References

1. Shi W, Jantsch M, Aljabar P, Pizarro L, Bai W, Wang H, et al. Temporal sparse free-form deformations. Medical image analysis. 2013;17(7):779–789.
