# Supplementary material for: A publicly available virtual cohort of four-chamber heart meshes for cardiac electro-mechanics simulations
Source: PLoS One. 2020 Jun 26;15(6):e0235145. doi: 10.1371/journal.pone.0235145 (PMC7319311; doi:10.1371/journal.pone.0235145)
Supplement: S1 Appendix — (PDF) [file pone.0235145.s008.pdf]

Appendix: Curvature and Wall Thickness Variability

We quantified the variability of left-ventricular wall thickness and local curvature of the endocardium of the four cardiac chambers and the intraventricular septum. We assigned the LV endocardium with a value for local wall thickness. Results are shown in Figure 1A. Our results show that there is a significant variability of LV wall thickness in the cohort. We found an even bigger variability within the same patient, meaning that wall thickness varies locally. The automatic segmentation tool failed to segment precisely the LV myocardium for cases 13 and 21. We therefore dilated the LV blood pool of a constant wall thickness to obtain the LV myocardium. Therefore, the boxplots for these cases show a much smaller LV wall thickness variability compared to the other cases.

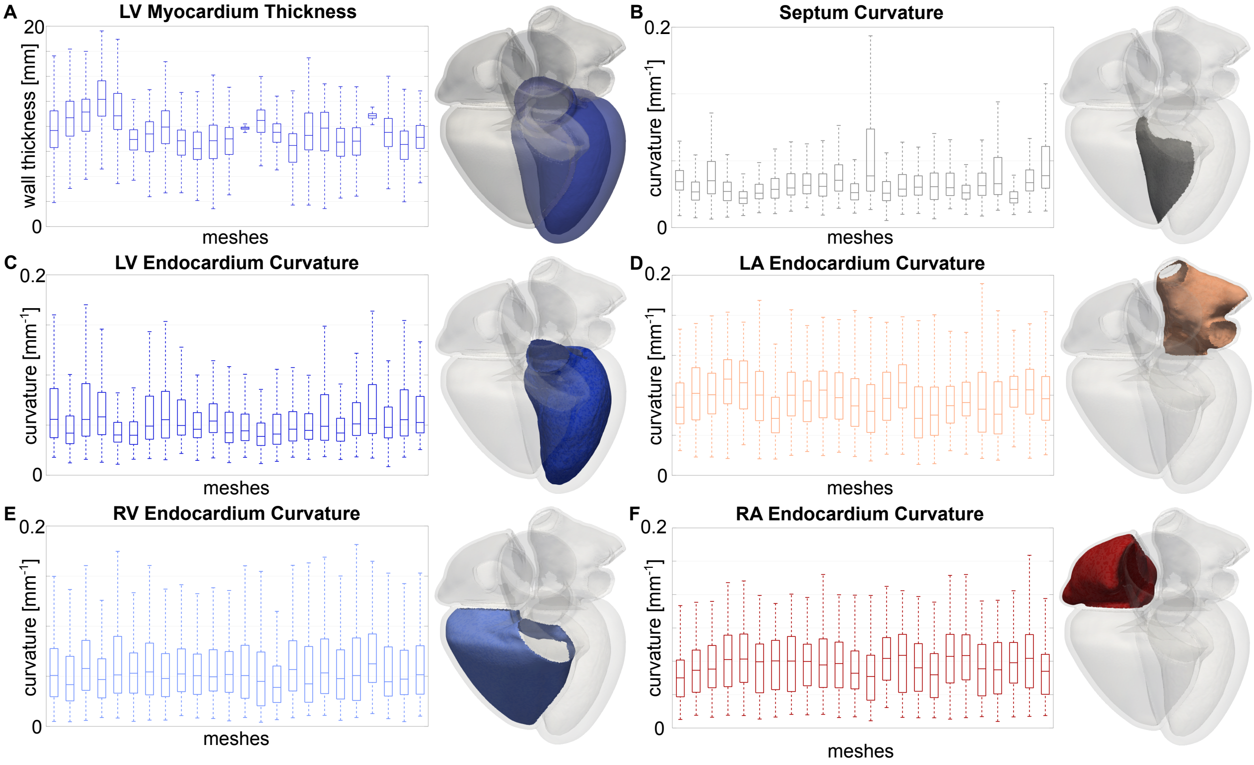

Fig. 1. LV myocardium thickness and endocardial curvature. A LV myocardium wall thickness. **B** Intraventricular septum curvature. **C** LV endocardium curvature. **D** LA endocardium curvature. **E** RV free wall endocardium curvature. **F** RA endocardium curvature. For the purpose of visualisation, we removed the outliers from the boxplots in figures B-F.

We computed local curvature distribution of the endocardial surface of the LV, RV, intraventricular septum, LA and RA. For each surface triangle, we took a patch of 10 elements in radius and we estimated the curvature of the patch. Results are shown in Figure 1B-F. The curvature of the LV endocardial surface and the intraventricular septum undergo the most variability within the cohort. On the other hand, the RV free wall, the LV and the RA do not change significantly among the patients.
